# Lymphocyte signatures correspond to clinical phenotypes in autoimmune limbic encephalitis

**DOI:** 10.1093/braincomms/fcaf156

**Published:** 2025-04-18

**Authors:** Saskia Räuber, Andreas Schulte-Mecklenbeck, Kelvin Sarink, Christoph Müller, Manoj Mannil, Lisa Langenbruch, Andre Dik, Sumanta Barman, Christine Strippel, Marco Gallus, Kristin S Golombeck, Christina B Schroeter, Alice Willison, Christopher Nelke, Fatme Seval Ismail, Wolfram Schwindt, Norbert Goebels, Stjepana Kovac, Heinz Wiendl, Gerd Meyer zu Hörste, Thomas Duning, Michael Hanke, Tobias Ruck, Walter Heindel, Udo Dannlowski, Tim Hahn, Catharina C Gross, Sven G Meuth, Nico Melzer

**Affiliations:** Department of Neurology with Institute of Translational Neurology, University of Münster, Münster 48149, Germany; Department of Neurology, Medical Faculty and University Hospital Düsseldorf, Heinrich Heine University of Düsseldorf, Düsseldorf 40225, Germany; Department of Neurology with Institute of Translational Neurology, University of Münster, Münster 48149, Germany; Institute for Translational Psychiatry, University of Münster, Münster 48149, Germany; Department of Neurology with Institute of Translational Neurology, University of Münster, Münster 48149, Germany; Department of Clinical Radiology, University of Münster, Münster 48149, Germany; Department of Neurology with Institute of Translational Neurology, University of Münster, Münster 48149, Germany; Department of Neurology with Institute of Translational Neurology, University of Münster, Münster 48149, Germany; Department of Neurology, Medical Faculty and University Hospital Düsseldorf, Heinrich Heine University of Düsseldorf, Düsseldorf 40225, Germany; Department of Neurology with Institute of Translational Neurology, University of Münster, Münster 48149, Germany; Department of Neurology with Institute of Translational Neurology, University of Münster, Münster 48149, Germany; Department of Neurology with Institute of Translational Neurology, University of Münster, Münster 48149, Germany; Department of Neurology, Medical Faculty and University Hospital Düsseldorf, Heinrich Heine University of Düsseldorf, Düsseldorf 40225, Germany; Department of Neurology with Institute of Translational Neurology, University of Münster, Münster 48149, Germany; Department of Neurology, Medical Faculty and University Hospital Düsseldorf, Heinrich Heine University of Düsseldorf, Düsseldorf 40225, Germany; Department of Neurology, Medical Faculty and University Hospital Düsseldorf, Heinrich Heine University of Düsseldorf, Düsseldorf 40225, Germany; Department of Neurology with Institute of Translational Neurology, University of Münster, Münster 48149, Germany; Department of Neurology, Medical Faculty and University Hospital Düsseldorf, Heinrich Heine University of Düsseldorf, Düsseldorf 40225, Germany; Department of Neurology, Klinikum Vest, Academic Teaching Hospital of the Ruhr University Bochum, Recklinghausen 45657, Germany; Department of Clinical Radiology, University of Münster, Münster 48149, Germany; Department of Neurology, Medical Faculty and University Hospital Düsseldorf, Heinrich Heine University of Düsseldorf, Düsseldorf 40225, Germany; Department of Neurology with Institute of Translational Neurology, University of Münster, Münster 48149, Germany; Department of Neurology with Institute of Translational Neurology, University of Münster, Münster 48149, Germany; Department of Neurology with Institute of Translational Neurology, University of Münster, Münster 48149, Germany; Department of Neurology with Institute of Translational Neurology, University of Münster, Münster 48149, Germany; Institute of Neuroscience and Medicine, Brain & Behaviour (INM-7), Research Centre Jülich, Jülich 52425, Germany; Institute of Systems Neuroscience, Medical Faculty and University Hospital Düsseldorf, Heinrich Heine University of Düsseldorf, Düsseldorf 40225, Germany; Department of Neurology with Institute of Translational Neurology, University of Münster, Münster 48149, Germany; Department of Neurology, Medical Faculty and University Hospital Düsseldorf, Heinrich Heine University of Düsseldorf, Düsseldorf 40225, Germany; Department of Clinical Radiology, University of Münster, Münster 48149, Germany; Institute for Translational Psychiatry, University of Münster, Münster 48149, Germany; Institute for Translational Psychiatry, University of Münster, Münster 48149, Germany; Department of Neurology with Institute of Translational Neurology, University of Münster, Münster 48149, Germany; Department of Neurology with Institute of Translational Neurology, University of Münster, Münster 48149, Germany; Department of Neurology, Medical Faculty and University Hospital Düsseldorf, Heinrich Heine University of Düsseldorf, Düsseldorf 40225, Germany; Department of Neurology with Institute of Translational Neurology, University of Münster, Münster 48149, Germany; Department of Neurology, Medical Faculty and University Hospital Düsseldorf, Heinrich Heine University of Düsseldorf, Düsseldorf 40225, Germany

**Keywords:** limbic encephalitis, multidimensional flow cytometry, immune cells, differential diagnoses

## Abstract

Autoimmune limbic encephalitis is an inflammatory condition confined to the limbic system of the brain that is deemed to be due to a dysregulated immune response. However, the exact pathophysiological mechanisms remain elusive. Diagnosis of autoimmune limbic encephalitis currently relies on clinical consensus criteria. However, diagnostic workup can be challenging, potentially delaying treatment initiation associated with poor clinical outcomes. We retrospectively identified 640 patients (81 autoimmune limbic encephalitis, 148 relapsing-remitting multiple sclerosis, 197 Alzheimer’s disease, 67 frontotemporal dementia, 37 temporal lobe epilepsy with hippocampal sclerosis and 110 somatic symptom disorder patients). Applying multidimensional flow-cytometry together with novel computational approaches, we analysed the peripheral blood and cerebrospinal fluid immune cell profiles at different disease stages and performed correlations with clinical parameters (i.e. neuropsychological performance, EEG and MRI). We were able to identify a shared immune signature of autoimmune limbic encephalitis showing similarities in adaptive B and T cell response with other inflammatory central nervous system diseases and in T cell patterns with neurodegenerative disorders. Antibody-negative autoimmune limbic encephalitis showed a pronounced T cell response in peripheral blood similar to temporal lobe epilepsy and hippocampal sclerosis and neurodegenerative disorders differentiating from antibody-positive autoimmune limbic encephalitis and classical inflammatory central nervous system diseases with regard to B and plasma cell response. Longitudinal immune cell phenotyping in autoimmune limbic encephalitis revealed dynamic changes over time mainly affecting the innate, B and plasma cell compartment. Correlation analysis indicated associations between the baseline immune cell profile, especially lymphocytes, and neuropsychological performance, as well as EEG and MRI abnormalities. Applying novel computational approaches, we found that multidimensional flow cytometry together with routine CSF parameters could reliably distinguish autoimmune limbic encephalitis from controls and clinical differential diagnoses. Incorporation of multidimensional flow cytometry parameters showed superior discriminatory ability compared with CSF routine parameters alone. Taken together, autoimmune limbic encephalitis is characterized by a B and T cell dominated intrathecal immune-cell signature corresponding to changes reported in the brain parenchyma and showing similarities with classical inflammatory central nervous system diseases and neurodegenerative disorders. Incorporating clinical parameters and applying novel computational approaches, we could show that multidimensional flow cytometry might be a beneficial complement to the established diagnostic workup of autoimmune limbic encephalitis promoting early diagnosis and facilitating outcome prediction to enhance individualized treatment regimes.

## Teaser

Räuber *et al*. report that autoimmune limbic encephalitis features a pronounced peripheral T and intrathecal T, B, and plasma cell response. Correlation with clinical parameters supports the pathophysiological relevance of immune cell subsets. Immune cell profiles show diagnostic potential and might complement the established diagnostic workup of autoimmune limbic encephalitis.

## Graphical Abstract

**Graphical Abstract:**
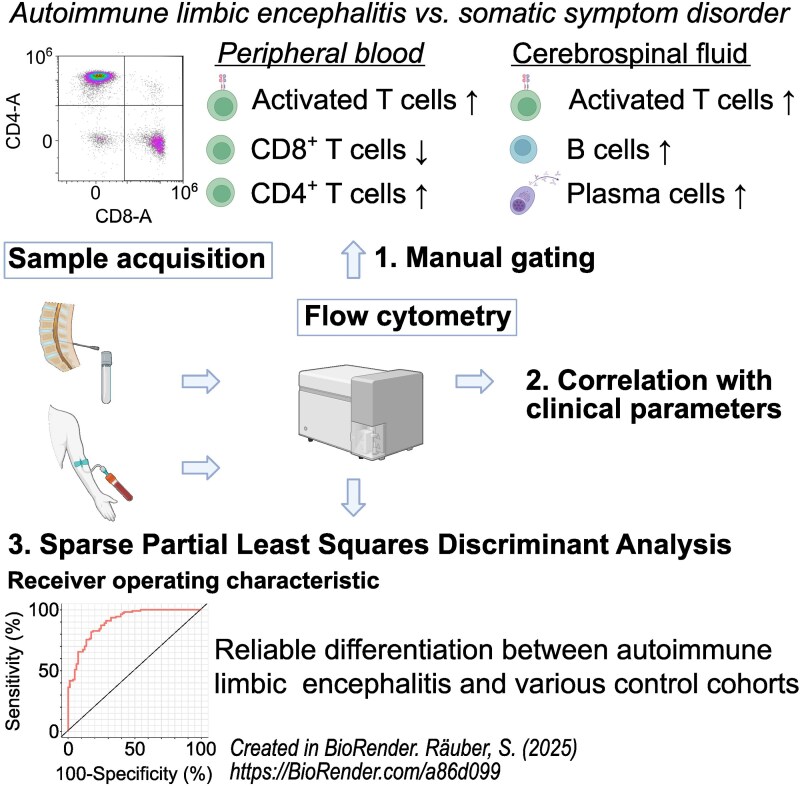

## Introduction

Autoimmune limbic encephalitis (ALE) is an inflammatory condition confined to the limbic system that is thought to be due to a dysregulated immune response.^1,2^ The most common forms of ALE are associated with autoantibodies (AABs) against leucine-rich glioma inactivated 1 (anti-LGI1 ALE) and the 65 kDa isoform of the glutamic acid decarboxylase (anti-GAD65 ALE) while the most common form of autoimmune encephalitis in general is anti-N-methyl-D-aspartate receptor encephalitis. Typical symptoms of ALE include subacute-onset temporal lobe seizures, memory deficits and behavioural changes consistent with an involvement of the limbic system.^1^ Graus *et al*.^1^ suggested recently validated diagnostic criteria based on clinical manifestation, MRI, EEG, routine CSF analysis and detection of AABs targeting neural antigens (referred to as antibody-positive ALE).^3^ On MRI, signal alterations in the medial temporal lobes on T2-weighted fluid-attenuated inversion recovery images are characteristic of ALE. EEG typically exhibits epileptic or slow-wave activity involving the temporal lobes. Routine CSF studies can show pleocytosis, an elevated immunoglobulin G (IgG) index or CSF-specific oligoclonal bands (ocbs). However, CSF analysis remains inconclusive in certain cases.^1^ In some patients, especially those without inflammatory routine CSF findings and without detectable AABs in serum and CSF (referred to as antibody-negative ALE), clinical diagnosis can be challenging leading to diagnostic and consecutively also therapeutic delay.^4,5^ However, an early treatment initiation is associated with better outcomes of autoimmune encephalitis patients^6-9^

Over the last years, technical and conceptual advances led to a more concise clinical and pathophysiological understanding of ALE and facilitated the detection of an increasing number of AABs in serum and/or CSF.^10^ AABs can target cell surface neural antigens (e.g. LGI1, CASPR2, GABA-B-, AMPA- and glycine receptor) or intracellular neural antigens (e.g. Hu, Ri, Ma2, amphiphysin, drebrin and GAD65) and prevailing immune effector mechanisms in the brain parenchyma differ based on the cellular localization of the targeted antigens^2,11-13^ A previous study analysed brain tissue from a small number of patients with autoimmune encephalitis and found a pivotal role for T cell-mediated neuronal cytotoxicity in patients with AABs against intracellular antigens.^14^ A follow-up study of patients with anti-GAD65 TLE confirmed the infiltration of CD8^+^ cytotoxic T cells (Tc) and detected high numbers of plasma cells (Pc) in the beginning of the disease course. However, no signs of antibody-mediated tissue damage were visible.^15^ Even though those finding indicate a central role of the lymphocyte-driven immune response in the pathogenesis of ALE, especially in ALE with AABs targeting intracellular antigens, sample sizes of histological analyses remain small due to the limited availability of brain parenchyma specimen. In turn, multidimensional flow cytometry (mFC) of PB and CSF facilitates broad characterization of immune cell populations providing insights into the local pathology of the CNS.^16^ Furthermore, correlation of immune cell populations with clinical parameters is suitable to assess their diagnostic and therapeutic implications. PB mFC analysis from patients with TLE and TLE with ALE (TLE-ALE) revealed an increased CD4^+^/CD8^+^ Tc ratio in PB of the TLE-ALE group further supporting the relevance of the Tc response.^17^ In addition, antibody-negative ALE patients showed higher proportions of B cells (Bc) in CSF and PB in relation to ALE patients with AABs targeting intracellular antigens.^18^ CSF analysis of patients with anti-GABA_B_-R ALE revealed increased amounts of Bc and Pc as well as CD4^+^ and CD8^+^ Tc. Correlation of immune cell profiles with the neuropsychological performance indicated an association between Pc, activated CD8^+^ Tc and neuropsychological outcomes.^11^ Overall, this hints towards a specific shift in immune cell profiles of ALE patients indicating diagnostic and prognostic potential. However, clinical and immunological characterization of a sizable cohort of immunotherapy-naive ALE patients with implications on immunopathogenesis, diagnosis and clinical outcome has not been previously performed.

We here provide a broad analysis of PB and CSF immune cell profiles of 81 mainly immunotherapy-naive ALE patients in comparison to 110 non-inflammatory and 148 inflammatory controls as well as 301 clinical differential diagnoses using mFC and novel computational approaches. Combining clinical parameters and mFC data, acquired at different disease stages, we assessed (i) whether mFC could aid the diagnosis of ALE, especially in cases without detectable AABs, promoting early diagnosis and treatment and (ii) whether mFC at baseline could be used as a prognostic tool facilitating personalized therapeutic approaches to improve outcomes.

## Material and methods

The local database of the Department of Neurology with Institute of Translational Neurology at the University Hospital Münster, Germany was queried to retrospectively identify patients who have been diagnosed with ALE since 2012. All patient charts were reviewed, and only patients who fulfilled the diagnostic criteria suggested by Graus *et al*.^1^ (*n* = 81) were included. Based on these criteria,^1^ patients were divided into different panels (Table 1).

**Table 1:** Diagnostic criteria of autoimmune limbic encephalitis according to Graus *et al*.^1^

Possible ALE (panel 1)	Definite ALE (panel 2)	Autoantibody-negative but probable ALE (panel 7)
Subacute onset (<3 months) of memory deficits, psychiatric symptoms or altered mental status At least one other typical feature: new focal CNS signs seizures (not explained by another known disorder) CSF pleocytosis hyperintense signal on FLAIR MRI highly restricted to one or both medial temporal lobes Exclusion of reasonable differential diagnoses	Subacute onset (<3 months) of memory deficits, psychiatric symptoms or seizures Hyperintense signal on FLAIR MRI highly restricted to both medial temporal lobes At least one: CSF pleocytosis EEG with epileptiform or slow-wave activity involving the medial temporal lobes of both hemispheres Exclusion of reasonable differential diagnoses	Subacute onset (<3 months) of memory deficits, psychiatric symptoms or altered mental status No detectable AABs in serum or CSF + at least two other typical features: CSF pleocytosis, elevated CSF IgG index or CSF-specific ocbs, or both Hyperintense signal on FLAIR MRI sequences highly restricted to one or both medial temporal lobes brain biopsy showing inflammatory infiltrates and excluding other disorders Exclusion of reasonable differential diagnoses

AABs, autoantibodies; ALE, autoimmune limbic encephalitis; CSF, cerebrospinal fluid; EEG, electroencephalography; FLAIR, T2-weighted fluid-attenuated inversion recovery; Ig, immunoglobulin; MRI, magnetic resonance imaging; ocbs, oligoclonal bands.

The AAB testing was performed using a combination of tissue-based assays, cell-based assays and immunoblotting (IB) (TBA + cell-based assays or TBA + IB). The following target antigens were tested: Hu, Ri, ANNA-3, Yo, Tr/DNER, Myelin, Ma/Ta, GAD65, Amphiphysin, Aquaporin-4, NMDA receptor, AMPA receptor, GABA-A/B receptor, LGI1, CASPR2, ZIC4, DPPX, glycine receptor, mGluR1, mGluR5, Rho-GTPase activating protein 26, ITPR1, Homer 3, MOG, Neurochondrin, GluRD2, Flotillin 1/2, IgLON5, CV2, Recoverin, SOX1, Titin, VGKC, Neurexin-3α and GFAP.

None of the patients received any immunotherapy at baseline sampling. Patients treated with any immunosuppressants (except for steroids) prior to sampling were excluded.

Additionally, we identified a large cohort of inflammatory and non-inflammatory control patients (see Supplementary Methods). All patient groups received routine CSF analysis as well as mFC of the PB and CSF. For the ALE cohort, EEG, MRI and neuropsychological data, which were collected during clinical routine workup, were retrospectively analysed. All data were collected ± 3 months around the neuropsychological assessment. For parts of the ALE cohort, follow-up data were available on average 13.3 ± 7.7 months after the baseline visit (Supplementary Fig. 1). About 66.7% of ALE patients have not been treated with long-term immunomodulatory therapy prior to follow-up. About 5.3% have received methotrexate, 19.3% rituximab, 1.8% methotrexate and rituximab, 1.8% rituximab and azathioprine, 1.8% cyclophosphamide, 1.8% azathioprine and cyclophosphamide and 1.8% cisplatin and etoposide. All methods including the statistical analysis are described in detail in the Supplementary Methods.

### Data analysis, visualization and statistical analysis

Data analysis and visualization were performed with ‘R studio’ (2023.06.1) and ‘GraphPad Prism’ (version 9.0.0). R packages ‘umap’ (v0.2.10.0) and ‘ggplot2’ (v3.4.4) were used to create UMAPs (uniform manifold approximation and projection for dimension reduction) of scaled and centered data. In order to create heatmaps, medians of the percentages of the different cell populations were calculated using Excel. The results were loaded into R Studio and were converted into a data frame. Next, data were scaled and centered by applying the function ‘scale’. Centering was done by subtracting the column medians from each value in that column. Next, scaling was performed by dividing each centered value by the standard deviation of the respective column. Finally, the centered and scaled data were plotted as a heatmap using the R package ‘pheatmap’ (v1.0.12). Violin plots with overlaying boxplots and volcano plots were created with the R package ‘ggplot2’ (v3.4.4). *P*-values were calculated using ANOVA with *post hoc* Tukey HSD, if normality could be assumed based on Shapiro–Wilk test; otherwise, Kruskal–Wallis test with Dunn *post hoc* test was applied. For two groups, *t*-test or Mann–Whitney U-test was used. To adjust for multiple comparisons, the Benjamini–Hochberg procedure was performed. A *P*-value of <0.05 was considered statistically significant. To create volcano plots, the log2 fold change was computed for every parameter. *P*-values were plotted against the corresponding log2 fold change. All significant parameters are colored and labelled. Multiple linear regression was performed to adjust for differences in age and sex between groups. Parameters which did not remain significant after correcting for age and sex are shown in grey. Sparse Partial Least Squares Discriminant Analysis (sPLS-DA) was used to assess the performance of the PB mFC, CSF routine and CSF mFC parameters to differentiate between ALE and control groups. SPLS-DA was performed using the R package ‘mixOmics’ (v6.26.0). The ‘auroc’ function was applied to calculate the area under the curve (AUC) for the classification results obtained from sPLS-DA. The resulting AUC values range from 0.5 (uninformative) to 1.0 (perfect). Furthermore, the contribution of the top 10 variables on latent component 1 was visualized.

Correlation analyses were performed with ‘R Studio’ using Pearson correlation coefficient if normality could be assumed based on D’Agostino & Pearson test. Otherwise, Spearman correlation coefficient was used.

## Results

### Patients’ characteristics and basic CSF parameters

In total, 640 patients were included. Basic cohort characteristics including CSF routine parameters are summarized in Table 2 and Supplementary Table 1. With regard to the ALE cohort, 61.7% of patients met the diagnosis of definite ALE, 34.6% of possible ALE and 3.7% of probable ALE according to Graus *et al.*^1^ (Table 2 and Supplementary Table 1). In 48.2% of ALE patients, antibodies targeting well-known antigens were detected, of which 51.3% had antibodies against cell surface neural antigens and 48.7% against intracellular neural antigens. About 49.4% of patients were antibody-negative, and two patients had antibodies against an unknown antigen. For 57 ALE patients, longitudinal follow-up data were available, on average 13.3 ± 7.7 months after baseline sampling.

**Table 2:** Basic cohort characteristics

	ALE	RRMS	AD	FTD	TLE-HS	SD
Number of patients	81	148	197	67	37	110
Age (mean ± STD) [years]	55.1 ± 14.5	32.0 ± 10.4	70.1 ± 9.7	63.8 ± 9.0	47.2 ± 15.7	39.8 ± 16.9
Sex [% male]	56.8	27.0	43.2	70.1	62.2	30.9
AABs in serum and/or CSF [% of patients]	48.2 (cs-t-ags^a^: 51.3; ic-t-ags^b^: 48.7)	n/a	n/a	n/a	n/a	n/a
Classification based on Graus *et al*. [% of patients]	Definite ALE: 61.7 Probable ALE: 3.7 Possible ALE: 34.6	n/a	n/a	n/a	n/a	n/a
Immunotherapy within 4 weeks prior to sampling [% of patients]	Plex: 1.2 Steroids: 17.3	0	0	0	0	0
ASM/sedative drugs at baseline [% of patients]	64.2/11.1	n/a	n/a	n/a	n/a	n/a
CSF routine—WBC (mean ± STD) [/µL]	2.29 ± 3.76	7.49 ± 9.68	0.74 ± 0.86	0.76 ± 0.95	1.27 ± 1.98	0.65 ± 0.84
CSF routine—BCSFBD [% of patients]	26.3	19.1	16.2	29.9	32.4	0
CSF routine—ocbs [% of patients]	13.8	86.9	2.1	1.5	0	0

^a^LGI1 = 11, CASPR2 = 3, GABA-B-R = 2, GABA-A-R = 1, DPPX = 1, VGKC = 1, Neurexin-3α = 1 patient(s).

^b^GAD65 = 11, Hu = 2, Ma/Ta = 1, Yo/Ma/Ta = 1, Hu/Sox/ZIC4/Yo = 1, GFAP = 1 patient(s).

AABs, autoantibodies; ALE, autoimmune limbic encephalitis; ASM, anti-seizure medication; BCSFBD, blood–CSF barrier dysfunction; CSF, cerebrospinal fluid; cs-t-ags, cell surface neural target antigens; FTD, frontotemporal dementia; ic-t-ags, intracellular neural target antigens; n/a, not applicable; ocbs, oligoclonal bands; plex, plasmapheresis; RRMS, relapsing remitting multiple sclerosis; SD, somatic symptom disorder; STD, standard deviation; WBC, white blood cell count.

### Changes in PB T cell profiles in ALE and neurodegenerative disorders compared with SD controls

**Figure 1:**
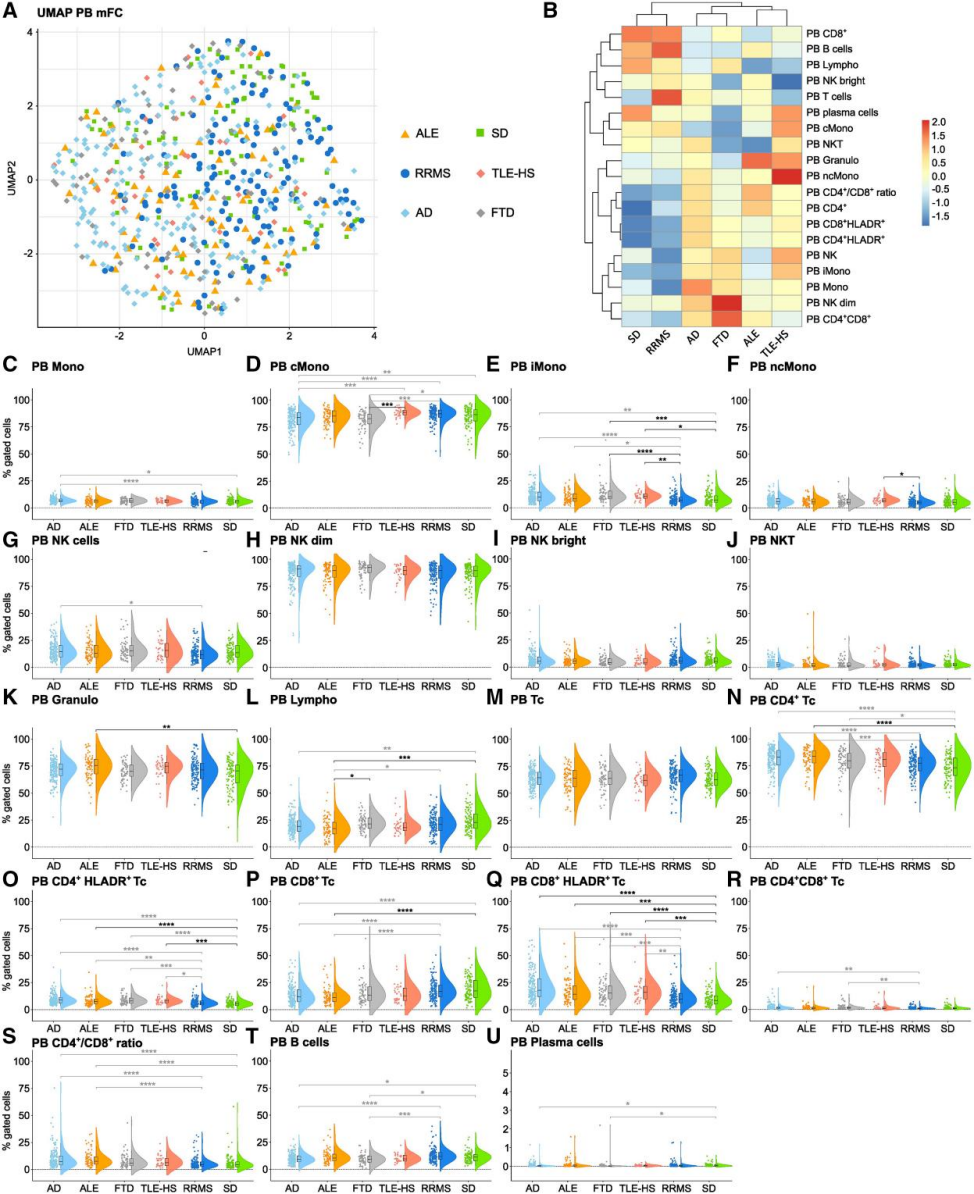
**Alterations in the peripheral T cell response in ALE and neurodegenerative disorders compared with SD controls**. (**A**) UMAP analysis including peripheral blood mFC parameters of patients with ALE, RRMS, TLE-HS, Alzheimer’s disease, FTD and SD; (**B**) Heatmap analysis of peripheral blood mFC parameters: the median of each parameter was calculated, centred, scaled, and clustered hierarchically; (**C-U**) Violin plots with box plots illustrating the peripheral blood mFC parameters of ALE, RRMS, TLE-HS, Alzheimer’s disease, FTD and SD patients. Medians and 25th as well as 75th percentiles are displayed by boxes. The whiskers extend to the smallest and largest values (maximum: 1.5 * IQR from the hinge). ANOVA with *post hoc* Tukey HSD was used to calculate *P*-values if normality of data could be assumed; otherwise, *P*-values were calculated using Kruskal–Wallis test with Dunn *post hoc* test (*P*-adjustment method: Benjamini–Hochberg). *P ≤ 0.05, **P ≤ 0.01, ***P ≤ 0.001, ****P ≤ 0.0001. ALE, autoimmune limbic encephalitis; Bc, B cells; cMono, classical monocytes; FTD, frontotemporal dementia; Granulo, granulocytes; iMono, intermediate monocytes; Lympho, lymphocytes; mFC, multidimensional flow cytometry; Mono, monocytes; ncMono; non-classical monocytes; NK, natural killer cells; NKT, Natural killer T cells; PB, peripheral blood; RRMS, relapsing remitting multiple sclerosis; SD, somatic symptom disorder; Tc, T cells; TLE-HS, temporal lobe epilepsy and hippocampal sclerosis; UMAP, uniform manifold approximation and projection for dimension reduction.

MFC data from ALE patients were analysed and compared with data from RRMS patients—serving as prototypical autoimmune inflammatory CNS disease, SD as non-inflammatory control group and clinically relevant differential diagnoses [temporal lobe epilepsy and hippocampal sclerosis (TLE-HS), Alzheimer’s disease and FTD]. Uniform Manifold Approximation and Projection for Dimension Reduction analysis including PB mFC parameters revealed similar overall immune-cell signatures in the PB of patients with ALE, RRMS, TLE-HS, Alzheimer’s disease, FTD and SD (Fig. 1A).

In the PB, ALE patients had lower percentages of lymphocytes with a shift from CD8^+^ Tc to CD4^+^ Tc and an increase in activated lymphocytes (Fig. 1B, L, N–Q and S) compared with SD controls. RRMS patients showed no significant differences in the peripheral immune cell profile compared with SD controls (Fig. 1B–U). Comparing ALE with RRMS patients, the same differences in lymphocyte populations, which were seen between ALE patients and SD controls were observed (Fig. 1B, L, N–Q and S). Given the differences in age and sex between the ALE and RRMS cohort, several parameters were confounded by age and sex.

TLE-HS patients had higher percentages of iMono and activated Tc in the PB in comparison to SD controls (Fig. 1B, E, O and Q) while no differences could be noted compared with patients with ALE.

Patients with FTD showed a shift from cMono to iMono in the PB as well as an increase in CD4^+^ Tc and activated Tc compared with SD controls (Fig. 1B, D, E, N, O and Q). In turn, Bc and Pc were reduced in FTD patients (Fig. 1B, T and U). Due to the age and sex differences between groups, several parameters were confounded. When comparing ALE to FTD patients, only lymphocytes were lower in the PB of ALE than in FTD patients (Fig. 1B and L).

Patients with Alzheimer’s disease had a shift from cMono to iMono comparable to FTD patients in relation to SD controls. Furthermore, the overall percentage of monocytes was increased (Fig. 1B–E**)**. Regarding the adaptive immune response, similar changes in Tc subsets were observed as in ALE patients compared with SD controls (Fig. 1B, L, N–Q and S). In addition, Bc and Pc were reduced similar to FTD patients in comparison to SD controls (Fig. 1B, T and U). Again, relevant confounding of multiple parameters by differences in age and sex between groups was seen. No significant differences in the PB immune cell profile were noted when directly comparing ALE and patients with Alzheimer’s disease.

In summary, ALE patients were characterized mainly by alterations in the peripheral Tc response compared with SD controls, which were not observed in other inflammatory CNS diseases (i.e. RRMS). Patients with TLE-HS, FTD and Alzheimer’s disease showed similar PB Tc responses to ALE patients and differed in Bc and Pc responses and monocyte subsets.

### ALE shares intrathecal lymphocyte patterns with inflammatory CNS diseases and neurodegenerative disorders

**Figure 2:**
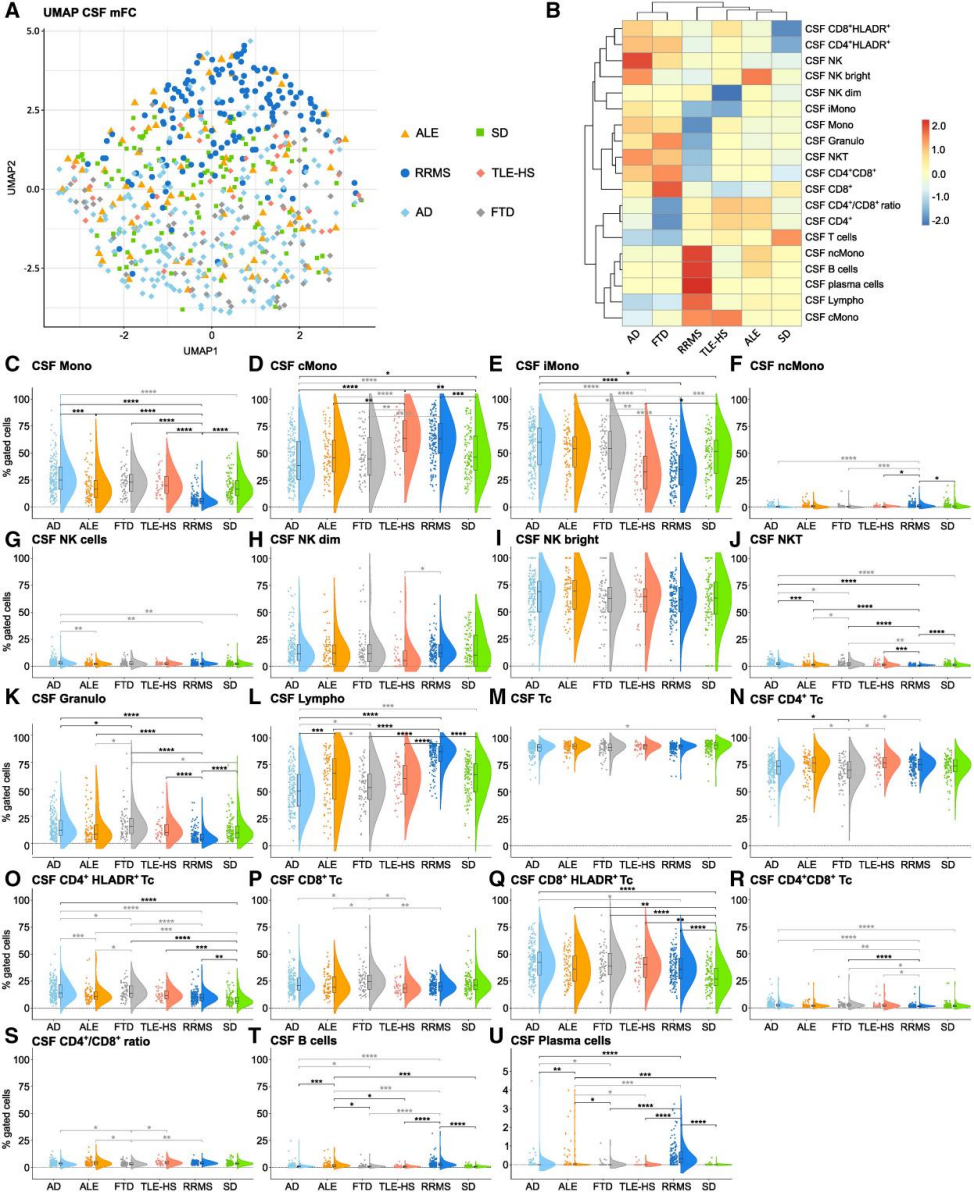
**ALE and RRMS share a pronounced intrathecal T and B cell response while similarities in T cell patterns were visible between ALE, TLE-HS, Alzheimer’s disease and FTD**. (**A)** UMAP analysis including cerebrospinal fluid mFC parameters of ALE, RRMS, TLE-HS, Alzheimer’s disease, FTD, and SD patients; (**B**) Heatmap analysis of cerebrospinal fluid mFC parameters: the median of each parameter was calculated, centred, scaled and clustered hierarchically; (**C-U**) Violin plots with box plots illustrating the cerebrospinal fluid mFC parameters of patients with ALE, RRMS, TLE-HS, Alzheimer’s disease, FTD and SD. Medians and 25th as well as 75th percentiles are displayed by boxes. The whiskers extend to the smallest and largest values (maximum: 1.5 * IQR from the hinge). ANOVA with *post hoc* Tukey HSD was used to calculate *P*-values if normality of data could be assumed; otherwise, *P*-values were calculated using Kruskal–Wallis test with Dunn *post hoc* test (*P*-adjustment method: Benjamini–Hochberg). *P ≤ 0.05, **P ≤ 0.01, ***P ≤ 0.001, ****P ≤ 0.0001. ALE, autoimmune limbic encephalitis; Bc, B cells; cMono, classical monocytes; CSF, cerebrospinal fluid; FTD, frontotemporal dementia; Granulo, granulocytes; iMono, intermediate monocytes; Lympho, lymphocytes; mFC, multidimensional flow cytometry; Mono, monocytes; ncMono; non-classical monocytes; NK, natural killer cells; NKT, Natural killer T cells; RRMS, relapsing remitting multiple sclerosis; SD, somatic symptom disorder; Tc, T cells; TLE-HS, temporal lobe epilepsy and hippocampal sclerosis; UMAP, uniform manifold approximation and projection for dimension reduction.

We next had a closer look at the CSF immune cell profile. Uniform Manifold Approximation and Projection for Dimension Reduction analysis showed a slightly distinct overall immune cell profile of RRMS patients compared with all other groups, although overlapping with some ALE patients. CSF immune-cell composition of patients with ALE, TLE-HS, Alzheimer’s disease, FTD and SD displayed a marked overlap (Fig. 2A). To assess significant differences in single parameters between groups, we performed heatmap analysis and created violin plots. ALE patients were characterized by an increase in activated Tc, in Bc and Pc in relation to SD controls (Fig. 2B, O, Q, T and U). Differences in CD4^+^HLADR^+^ Tc did not remain significant after correction for age and sex. RRMS patients featured alterations in the innate immune response with a decrease in the percentage of monocytes and a shift from iMono to cMono and ncMono (Fig. 2B–F). Moreover, NKT and granulocytes were lower compared with SD controls (Fig. 2B, J and K**)**. IMono were confounded by differences in age and sex. Regarding adaptive immunity, percentages of lymphocytes, activated Tc, Bc and Pc were increased compared with SD controls (Fig. 2B, L, O, Q, T and U). Differences in innate immunity (except for ncMono) were also seen when directly comparing RRMS to ALE patients (Fig. 2B–F). Concerning the adaptive immune response, lymphocytes, CD4^+^CD8^+^ Tc, Bc and Pc were elevated in RRMS in comparison to ALE patients (Fig. 2B, L, R, T and U). Due to the age and sex differences between groups, some parameters were confounded.

TLE-HS patients showed a shift from cMono to iMono also seen in RRMS patients compared with SD controls and ALE patients (Fig. 2B, D and E). Similar to ALE patients, activated Tc were higher in TLE-HS patients than in SD controls whereas ALE patients had higher fractions of Bc and Pc than TLE-HS patients (Fig. 2B, O, Q, U and T). However, several differences between ALE and TLE-HS were confounded by age and sex.

FTD patients showed elevated percentages of NKT and granulocytes compared with SD controls and ALE patients. Concerning adaptive immunity, CD4^+^CD8^+^ Tc and activated Tc were higher in FTD patients than in SD controls (Fig. 2B, J, K, O, Q and R). CD4^+^HLADR^+^ were even higher in FTD than in ALE patients while lymphocytes, CD4^+^ Tc, CD4^+^/CD8^+^ ratio, Bc and Pc were higher in ALE than in FTD patients. In addition, CD8^+^ were elevated in FTD compared with ALE patients (Fig. 2B, L, N-P and S–U). Again, confounding of multiple parameters by age and sex was observed.

Changes in several innate an adaptive immune cell populations were noted between patients with Alzheimer’s disease and SD controls. Concerning innate immunity, an increase in monocytes with a shift from cMono to iMono and higher fractions of NK and NKT were observed (Fig. 2B–E, G and J). Regarding adaptive immunity, we found lower percentages of lymphocytes and Tc while CD4^+^CD8^+^ Tc and activated Tc were increased in Alzheimer’s disease compared with SD controls (Fig. 2B, L, O, Q and R). The same differences in innate immune response (except for monocyte subsets), lymphocytes and CD4^+^HLADR^+^ Tc were observed between patients with Alzheimer’s disease and ALE. Moreover, Bc and Pc were elevated in ALE compared with patients with Alzheimer’s disease (Fig. 2B, C, G, J, L, O, T and U).

Taken together, ALE and classical inflammatory CNS diseases (i.e. RRMS) shared elevated intrathecal fractions of activated Tc, of Bc and Pc, the latter being even higher in RRMS than in ALE patients. RRMS patients featured changes in the innate immune response, especially monocyte subsets, which were not visible when comparing ALE patients and SD controls. Furthermore, ALE, TLE-HS, Alzheimer’s disease and FTD show similarities in the intrathecal Tc response, especially in activated lymphocytes, while differences were noted with regard to Bc, Pc and the innate immune response.

### Antibody-negative ALE is characterized by a pronounced T cell response in the PB

**Figure 3:**
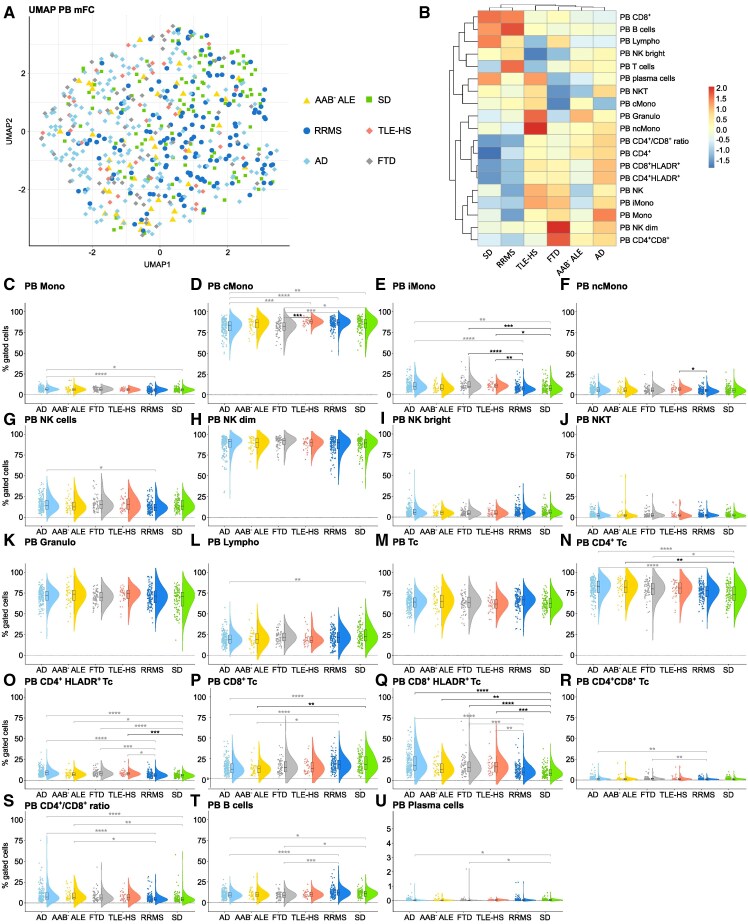
**Antibody-negative ALE shows a pronounced peripheral T cell response similar to TLE-HS, and neurodegenerative disorders**. (**A**) UMAP analysis including peripheral blood mFC parameters of patients with antibody-negative ALE, RRMS, TLE-HS, Alzheimer’s disease, FTD and SD; (**B**) Heatmap analysis of peripheral blood mFC parameters: the median of each parameter was calculated, centred, scaled and clustered hierarchically; (**C-U**) Violin plots with box plots illustrating the peripheral blood mFC parameters of patients with antibody-negative ALE, RRMS, TLE-HS, Alzheimer’s disease, FTD and SD. Medians and 25th as well as 75th percentiles are displayed by boxes. The whiskers extend to the smallest and largest values (maximum: 1.5 * IQR from the hinge). ANOVA with *post hoc* Tukey HSD was used to calculate *P*-values if normality of data could be assumed, otherwise *P*-values were calculated using Kruskal–Wallis test with Dunn *post hoc* test (*P*-adjustment method: Benjamini–Hochberg). *P ≤ 0.05, **P ≤ 0.01, ***P ≤ 0.001, ****P ≤ 0.0001. AAB^−^ ALE, autoantibody-negative ALE; ALE, autoimmune limbic encephalitis; cMono, classical monocytes; CSF, cerebrospinal fluid; FTD, frontotemporal dementia; Granulo, granulocytes; iMono, intermediate monocytes; Lympho, lymphocytes; mFC, multidimensional flow cytometry; Mono, monocytes; ncMono; non-classical monocytes; NK, natural killer cells; NKT, natural killer T cells; PB, peripheral blood; RRMS, relapsing remitting multiple sclerosis; SD, somatic symptom disorder; TLE-HS, temporal lobe epilepsy and hippocampal sclerosis; UMAP, uniform manifold approximation and projection for dimension reduction.

**Figure 4:**
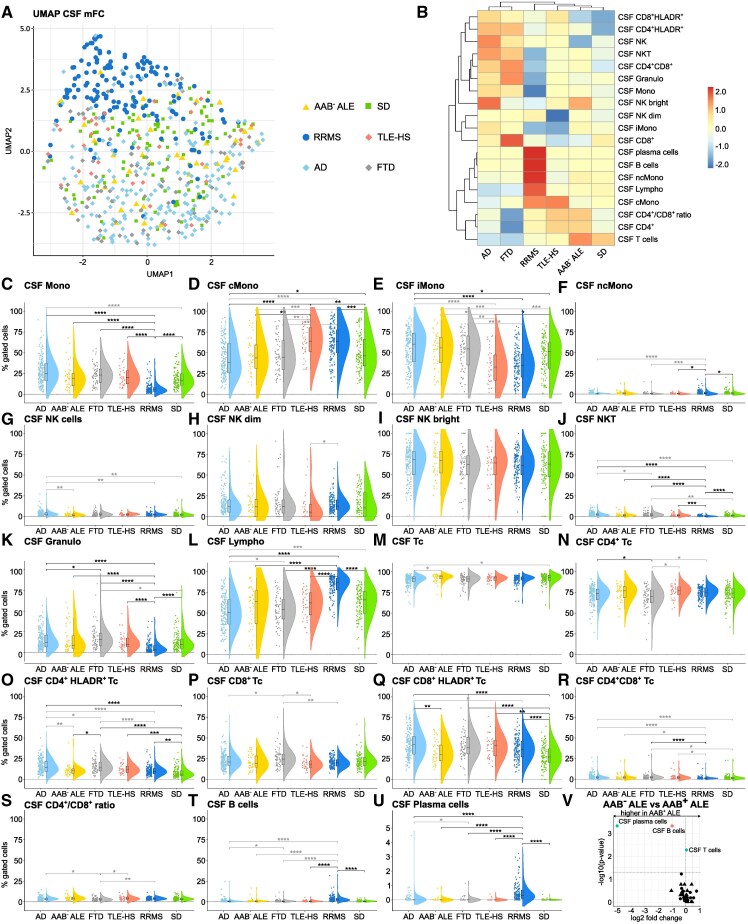
**Differences in the intrathecal innate and adaptive immune response between patients with antibody-negative ALE, RRMS, TLE-HS, Alzheimer’s disease and FTD**. (**A**) UMAP analysis including cerebrospinal fluid mFC parameters of patients with antibody-negative ALE, RRMS, TLE-HS, Alzheimer’s disease, FTD and SD; (**B**) Heatmap analysis of cerebrospinal fluid mFC parameters: the median of each parameter was calculated, centred, scaled and clustered hierarchically; (**C-U**) Violin plots with box plots illustrating the cerebrospinal fluid mFC parameters of patients with antibody-negative ALE, RRMS, TLE-HS, Alzheimer’s disease, FTD and SD. Medians and 25th as well as 75th percentiles are displayed by boxes. The whiskers extend to the smallest and largest values (maximum: 1.5 * IQR from the hinge). ANOVA with *post hoc* Tukey HSD was used to calculate *P*-values if normality of data could be assumed; otherwise, *P*-values were calculated using Kruskal–Wallis test with Dunn *post hoc* test (*P*-adjustment method: Benjamini–Hochberg). *P ≤ 0.05, **P ≤ 0.01, ***P ≤ 0.001, ****P ≤ 0.0001. (**V**) Volcano plot comparing the peripheral blood and cerebrospinal fluid mFC parameters of antibody-negative ALE and antibody-positive ALE patients. The fold change of every single parameter between the two groups is plotted against the corresponding *P*-value calculated by *t*-test if normality could be assumed based on the Shapiro–Wilk test; otherwise, Mann–Whitney U-test was used. To adjust for multiple comparisons, the Benjamini–Hochberg procedure was performed. PB parameters are shown as triangles and CSF parameters as circles. Only significant parameters are labelled. *P*-values < 0.0001 are depicted as 0.0001 and *P*-values > 0.9999 are shown as 1.0. AAB, autoantibody; ALE, autoimmune limbic encephalitis; Bc, B cells; cMono, classical monocytes; CSF, cerebrospinal fluid; FTD, frontotemporal dementia; Granulo, granulocytes; iMono, intermediate monocytes; Lympho, lymphocytes; mFC, multidimensional flow cytometry; Mono, monocytes; ncMono; non-classical monocytes; NK, natural killer cells; NKT, Natural killer T cells; RRMS, relapsing remitting multiple sclerosis; SD, somatic symptom disorder; Tc, T cells; TLE-HS, temporal lobe epilepsy and hippocampal sclerosis; UMAP, uniform manifold approximation and projection for dimension reduction.

As clinical diagnosis of antibody-negative ALE can be especially challenging, we repeated our analysis only including patients without detectable AABs in serum and CSF. Uniform Manifold Approximation and Projection for Dimension Reduction analysis of PB mFC parameters revealed a marked overlap between antibody-negative ALE, RRMS, TLE-HS, Alzheimer’s disease, FTD and SD (Fig. 3A). Similar to all ALE patients compared with SD controls, antibody-negative ALE patients featured a shift from CD8^+^ to CD4^+^ Tc in the PB and an increased fraction of activated Tc (Fig. 3B, N–Q and S). The increase in activated Tc was shared between patients with antibody-negative ALE, TLE-HS, FTD and Alzheimer’s disease. With regard to the CSF, Uniform Manifold Approximation and Projection for Dimension Reduction analysis indicated similarities between antibody-negative ALE, TLE-HS, Alzheimer’s disease and FTD distinct from RRMS patients and slightly different from SD controls (Fig. 4A). In contrast to the whole ALE cohort, antibody-negative ALE patients did not show an increase in activated Tc, in Bc and Pc in the CSF compared with SD controls (Fig. 4B, O, Q, T and U). When directly comparing antibody-negative and antibody-positive ALE patients, the latter showed higher percentages of CSF Bc and Pc, while antibody-negative ALE patients had higher fractions of CSF Tc (Fig. 4V).

Overall, antibody-negative ALE is characterized by changes in PB lymphocyte patterns with a shift from CD8^+^ to CD4^+^ Tc in the PB and a higher percentage of activated Tc. The latter were also increased in the PB of patients with TLE-HS, Alzheimer’s disease and FTD. In contrast, antibody-negative ALE patients differed from patients with TLE-HS, Alzheimer’s disease and FTD regarding the intrathecal Tc and innate immune response. Differences in PB Tc patterns as well as in the CSF Tc, Bc Pc, and innate immune response were noted between antibody-negative ALE and RRMS patients.

### Homogenous immune cell profiles amongst ALE subgroups

We further divided the ALE cohort into subgroups based on clinical and paraclinical parameters and compared PB/CSF mFC parameters between groups. To exclude a relevant confounding of steroid treatment, we first compared ALE patients who have received steroids prior to sampling and ALE patients who have not been treated with steroids before. The latter had higher percentages of lymphocytes and monocytes in PB while PB granulocytes were lower compared with patients previously treated with steroids. Apart from this, no other significant differences were found (Supplementary Fig. 2A).

Next, we divided our ALE cohort in definite ALE and possible/probable ALE according to Graus *et al*.^1^ No significant differences in PB or CSF immune cell profiles could be detected between those two groups (Supplementary Fig. 2B).

Moreover, we compared the immune cell profile of ALE patients with AABs targeting cell surface neural antigens and ALE patients with AABs against intracellular antigens. No differences were noted between those two groups (Supplementary Fig. 2C).

We further divided the ALE cohort into two groups based on unilateral or bilateral T2-fluid-attenuated inversion recovery hyperintensities on routine MRI. Immune cell composition was comparable between patients with uni- and bilateral MRI abnormalities (Supplementary Fig. 2D).

In summary, ALE shows a characteristic immune cell signature not affected by the clinical diagnosis based on current diagnostic consensus criteria, AAB target or lateralization of MRI abnormalities.

### Longitudinal phenotyping of ALE patients reveals dynamic changes over time

For a subgroup of ALE patients, longitudinal PB and CSF mFC data were available. Therefore, we compared PB and CSF immune cell profiles of ALE patients at follow-up with SD patients. Only ALE patients who have not been treated with long-term immunotherapies prior to follow-up sampling were included.

At follow-up, ALE patients had similar lymphocyte patterns in PB and CSF compared with baseline with a shift from CD8^+^ lymphocytes to CD4^+^ lymphocytes in PB and an increase in activated CD4^+^ lymphocytes in PB and CSF. In contrast to baseline, no significant differences were detected for activated CD8^+^ lymphocytes. Furthermore, ALE patients at follow-up did not display elevated fractions of CSF Bc and Pc but rather had lower percentages of PB B and Pc compared with SD. In addition, changes in innate immune populations (increase in CSF Mono, especially iMono, elevated CSF NKT and reduced PB NK dim) were visible at follow-up, which could not be detected at baseline (Supplementary Fig. 2E). Due to demographic imbalances between groups, several parameters were confounded by age and sex. Direct comparison between immune cell profiles at follow-up and at baseline supported the shift from an adaptive Tc, Bc and Pc-driven immune response at baseline to a pronounced innate immune response with persisting Tc effector mechanisms at follow-up (Supplementary Fig. 2F).

In summary, longitudinal analysis revealed dynamic changes in immune cell patterns in ALE over time with a persisting Tc response and a shift from a Bc and Pc response at baseline to an innate immune response at follow-up.

### Immune cell signature of ALE correlate with clinical parameters and might promote outcome prediction

We further incorporated clinical data in our analysis and first correlated the CSF routine and PB/CSF mFC parameters at baseline with performance on neuropsychological assessment (low scores illustrate poor performance on neuropsychological assessment). In the PB, numbers of NK cells positively and the percentage of Pc negatively correlated with figural memory *z*-scores (Supplementary Table 2A). The number of iMono as well as the percentage of iMono, CD4^+^ Tc and Pc had a negative correlation with verbal memory *z* scores. Furthermore, the number of NK cells, the percentage of cMono, NK cells and lymphocytes positively correlated with verbal memory z scores (Supplementary Table 2A). Moreover, a positive correlation was noted between the number of lymphocytes, Tc, (activated) CD8^+^ Tc, NK cells as well as the percentage of cMono, lymphocytes, CD8^+^ Tc and EpiTrack scores whereas a negative correlation was observed between the percentage of iMono, ncMono, granulocytes, CD4^+^, the CD4^+^/CD8^+^ ratio and EpiTrack scores (Supplementary Table 2A).

In the CSF, the white blood cell count (WBC), the number of different Tc subsets as well as the number and percentage of lymphocytes and Pc negatively correlated with figural memory *z* scores (Supplementary Table 2B). In addition, the WBC, the number of different Tc populations, Bc, Pc, as well as the percentage of ncMono and Pc had a negative correlation with verbal memory *z* scores (Supplementary Table 2B). Moreover, the WBC as well as the number and percentage of Pc negatively correlated with the EpiTrack score (Supplementary Table 2B). Concerning EEG and MRI abnormalities, the percentage of CD4^+^CD8^+^ Tc showed a negative correlation with the occurrence of interictal epileptiform discharges and slowing while the amount of CSF protein, the number and percentage of Pc and the percentage of Bc had a positive correlation with the mean fluorescence intensity (MFI) of the amygdalae (mean of both sides) on the MRI. Additionally, CSF NK cells negatively correlated with the MFI of the hippocampi (mean of both sides; Supplementary Table 2B).

Furthermore, antibody titres in CSF positively correlated with the CSF WBC, numbers and percentages of lymphocytes, Bc and Pc, as well as with the number of different Tc subsets and with MRI signal hyperintensities of the amygdalae (mean of both sides; Supplementary Table 2C).

We further correlated PB and CSF immune cell profiles at baseline with clinical parameters at follow-up. Regarding absolute cell numbers, we could detect positive correlations between PB NK cells, lymphocytes, CD8^+^ Tc and verbal memory *z* scores (Supplementary Table 2D). Moreover, positive correlations between the percentage of PB NK as well as Bc and figural memory *z* scores were observed (Supplementary Table 2D). The percentage of PB CD8^+^HLADR^+^, CSF CD4^+^HLADR^+^ and NK bright negatively correlated with verbal memory *z* scores while there was a positive correlation between the percentage of CSF NK dim and verbal memory *z* scores (Supplementary Table 2D). Only the amount of CSF protein at baseline correlated with interictal EEG abnormalities at follow-up (Supplementary Table 2D).

In summary, innate and adaptive immune cell populations at baseline correlate with performance on neuropsychological assessment, EEG and MRI abnormalities. Of note, higher amounts of lymphocytes in CSF are associated with poor memory performance and MRI signal hyperintensities of the amygdalae emphasizing their pathophysiological relevance. Assessment of immune cell profiles might be useful for outcome prediction facilitating identification of patients benefiting from an intensified treatment regimen.

### MFC might support the diagnostic workup of ALE

Diagnosis of ALE, especially of antibody-negative ALE, can be challenging, delaying diagnosis and treatment. However, early diagnosis and treatment is crucial for a good clinical outcome. We therefore applied novel computational approaches to assess the benefit of combining PB and CSF mFC with CSF routine analysis as part of the diagnostic workup of ALE. First, we assessed the discriminatory ability of CSF routine parameters using sPLS-DA. Receiver operating characteristic analysis yielded moderate results with an AUC of 0.817 (ALE versus SD), 0.662 (ALE versus TLE-HS), 0.707 (ALE versus Alzheimer’s disease), 0.740 (ALE versus FTD), 0.681 [ALE versus neurodegenerative disorders (NDD = TLE-HS, Alzheimer’s disease, FTD)], 0.814 (antibody-negative ALE versus SD), 0.632 (antibody-negative ALE versus TLE-HS), 0.735 (antibody-negative ALE versus Alzheimer’s disease), 0.694 (antibody-negative ALE versus FTD) and 0.674 (antibody-negative ALE versus NDD; Supplementary Fig. 3A–D, F–J and L). Only RRMS patients could be differentiated from (antibody-negative) ALE with high AUC when including CSF routine parameters in the model (Supplementary Fig. 3E and K).

**Figure 5:**
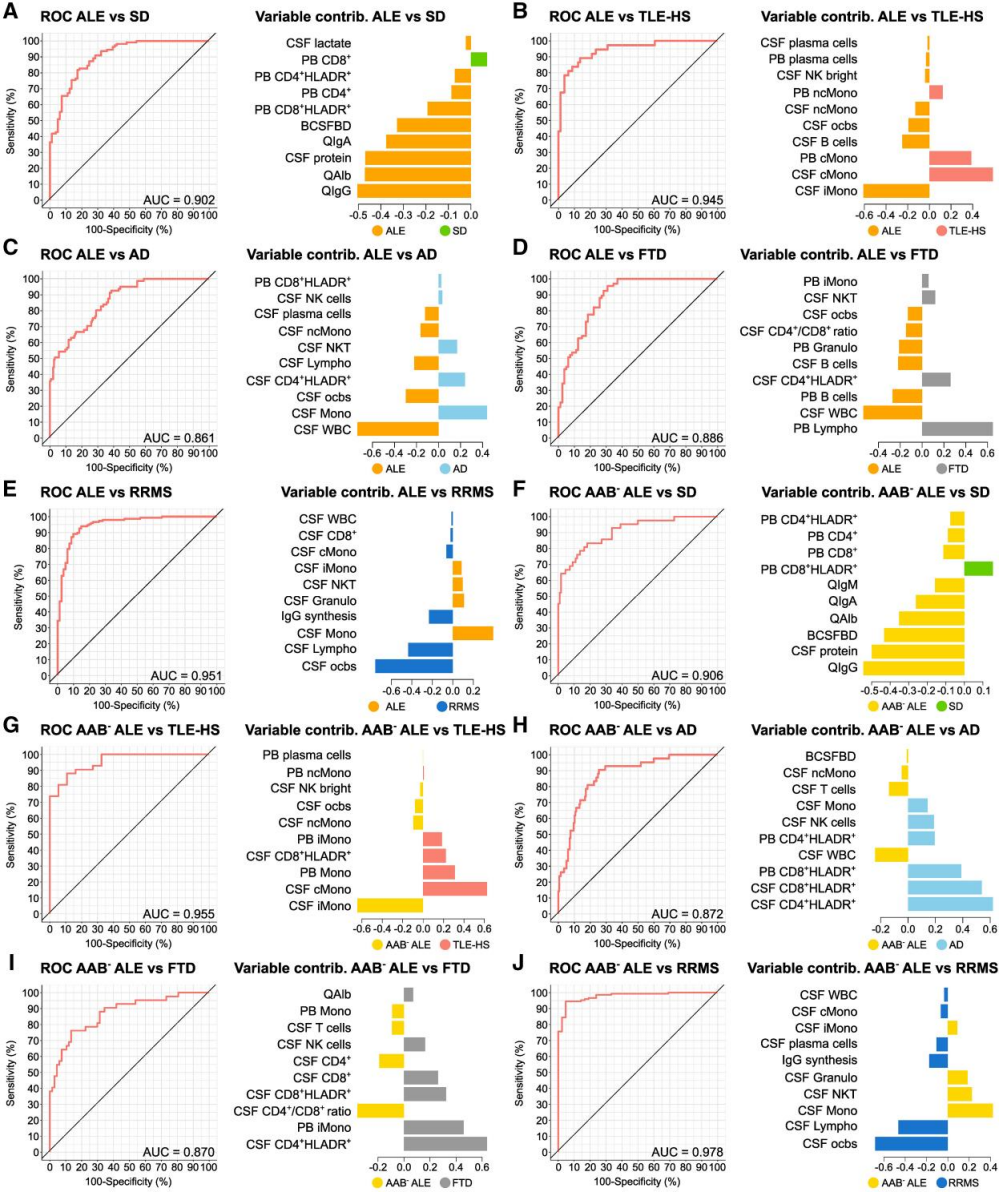
**MFC together with CSF routine analysis can reliably distinguish ALE from differential diagnoses and controls**. (**A-J**) ROC analyses of the classification results obtained from sPLS-DA. CSF routine as well as PB and CSF mFC parameters were included in the analysis. In addition, the contribution of the top 10 variables on latent component 1 was visualized. ALE patients (**A-E**) or antibody-negative ALE patients (**F-J**) were compared with one control cohort at a time. AAB^−^ ALE, autoantibody-negative ALE; AD, Alzheimer’s disease; ALE, autoimmune limbic encephalitis; AUC, area under the curve; BCSFBD, blood-CSF barrier dysfunction; cMono, classical monocytes; contrib. , contribution; CSF, cerebrospinal fluid; FTD, frontotemporal dementia; Granulo, granulocytes; Ig, immunoglobulin; iMono, intermediate monocytes; Lympho, lymphocytes; mFC, multidimensional flow cytometry; Mono, monocytes; ncMono; non-classical monocytes; NK, natural killer cells; NKT, natural killer T cells; PB, peripheral blood; ocbs, oligoclonal bands; QIgA, serum/CSF IgA ratio; QAlb, serum/CSF albumin ratio; QIgG, serum/CSF IgG ratio; QIgM, serum/CSF IgM ratio; ROC, receiver operating characteristic; RRMS, relapsing remitting multiple sclerosis; SD, somatic symptom disorder; sPLS-DA, Sparse Partial Least Squares Discriminant Analysis; TLE-HS, temporal lobe epilepsy and hippocampal sclerosis; WBC, white blood cell count.

We next combined CSF routine with PB and CSF mFC parameters and repeated the analysis. This time, ALE could be differentiated from SD with an AUC of 0.902. Serum/CSF IgG ratio (QIgG), albumin ratio and CSF protein had the highest variable contribution (Fig. 5A). Comparing ALE and TLE-HS, an AUC of 0.945 could be reached, CSF iMono, CSF cMono, and PB cMono being the most important variables (Fig. 5B). SPLS-DA could differentiate ALE and Alzheimer’s disease with an AUC of 0.861 with CSF WBC, CSF Mono and CSF ocbs contributing the most to the model (Fig. 5C). ALE could be distinguished from FTD with an AUC of 0.886. PB lymphocytes, CSF WBC and PB Bc were the most important parameters (Fig. 5D). An AUC of 0.951 was achieved when differentiating ALE from RRMS with CSF ocbs, CSF lymphocytes and monocytes being the most important parameters (Fig. 5E). As antibody-negative ALE is especially difficult to diagnose, we repeated the analysis only including this subgroup of ALE patients. Differentiating antibody-negative ALE and SD, sPLS-DA achieved a high AUC with 0.906. Variable contribution analysis revealed QIgG, CSF protein and blood-CSF barrier dysfunction to be the most important parameters (Fig. 5F). When comparing antibody-negative ALE and TLE-HS, both groups could be differentiated with an AUC of 0.955. CSF iMono, CSF cMono and PB Mono had the highest variable contribution (Fig. 5G). Antibody-negative ALE and Alzheimer’s disease as well as FTD could be distinguished with an AUC of 0.872 and 0.870, respectively. CSF CD4^+^HLADR^+^ and CSF/PB CD8^+^HLADR^+^ were the most relevant parameters when comparing antibody-negative ALE and Alzheimer’s disease, CSF CD4^+^HLADR^+^, PB iMono, CSF CD4^+^/CD8^+^ ratio when comparing antibody-negative ALE and FTD (Fig. 5H and I). An AUC of 0.978 was achieved when differentiating antibody-negative ALE and RRMS. CSF ocbs, lymphocytes and monocytes had the highest variable contribution (Fig. 5J). Finally, we combined patients with TLE-HS, Alzheimer’s disease and FTD (NDD), as similarities in clinical presentation make them relevant differential diagnoses of ALE. SPLS-DA could distinguish ALE from NDD with an AUC of 0.830, CSF WBC, ocbs and Pc being the most relevant parameters (Supplementary Fig. 3M). Antibody-negative ALE and NDD could be differentiated with an AUC of 0.834; CSF CD4^+^HLADR^+^, CD8^+^HLADR^+^ and PB CD8^+^HLADR^+^ had the highest variable contribution (Supplementary Fig. 3N).

Taken together, the combination of CSF routine with PB and CSF mFC parameters could reliably differentiate ALE from controls and clinically relevant differential diagnoses and showed superior ability to CSF routine parameters alone. Thus, mFC might facilitate early diagnosis and treatment of ALE likely improving long-term outcomes.

## Discussion

ALE presents a severely disabling autoimmune inflammatory condition of the limbic system, which became increasingly recognized due to technical and conceptual advances over the last years. Currently, diagnosis relies on clinical consensus criteria taking into account clinical manifestation, EEG, MRI and CSF routine parameters.^1^ However, diagnostic workup can be challenging in certain cases, especially if no AABs can be detected in serum and CSF.^4^ Bc and Tc driven immune responses seem to contribute to the local pathology in the CNS; however, the exact mechanisms remain insufficiently characterized to date.^2,12^ Previous studies highlighted the benefit of mFC to elucidate disease pathogenesis and assessed its diagnostic value^16-18^ However, immune phenotyping of a sizable cohort of treatment-naive ALE patients taking into account clinical parameters and follow-up data has not been previously performed.

Analysing PB and CSF immune cell profiles of a sizable cohort of mainly treatment-naive ALE patients in comparison to various inflammatory and non-inflammatory control groups, we were able to identify a shared immune signature of ALE showing similarities in the adaptive Bc and Tc response with inflammatory CNS diseases and in Tc patterns with neurodegenerative disorders. Antibody-negative ALE showed a pronounced Tc response in PB similar to TLE-HS and neurodegenerative disorders differentiating it from antibody-positive ALE and classical inflammatory CNS disease with regard to Bc and Pc response. Longitudinal immune cell phenotyping revealed dynamic changes over time mainly affecting the innate, Bc and Pc compartment. Correlation analysis with clinical parameters revealed associations between the immune cell profile at baseline and neuropsychological performance, MRI and EEG abnormalities at baseline and follow-up, which might facilitate outcome prediction and impact treatment regimes. Finally, our results indicate that mFC together with routine CSF parameters could be a valuable complementary tool to the existing diagnostic workup promoting early diagnosis of ALE.

Previous studies reported alterations in adaptive immunity in the CSF and brain parenchyma of ALE patients.^2,11,18,19^ Assessing the PB and CSF immune cell profile of a sizable cohort of mainly treatment-naive ALE patients by mFC, we could confirm a Bc- and Tc-driven immune response in ALE compared with non-inflammatory controls further supporting the significant involvement of the adaptive immune system in the pathogenesis of ALE.^11,17,18,20^ Comparison of immune cell populations between ALE, RRMS, TLE-HS, and neurodegenerative disorders (i.e. Alzheimer’s disease and FTD) identified similarities in adaptive immune cell patterns with an increase in activated CD4^+^ and CD8^+^ Tc in PB and/or CSF. A Tc-driven immune response has been described in inflammatory CNS diseases,^21^ as well as neurodegenerative disorders^22^ and TLE-HS.^23^ Our results hint towards shared pathophysiological mechanisms regarding adaptive immunity between classical inflammatory CNS diseases and primarily neurodegenerative disorders. Under physiological conditions, the CNS parenchyma is almost exclusively populated by resident microglia and myeloid cells.^24^ Following disruption of homeostasis—as seen in various different CNS disorders—Tc transmigrate the blood-brain-barrier and enter the brain parenchyma. In this scenario, they exert both inflammatory and anti-inflammatory functions depending on the subset and polarization.^25^ A regulated Tc response is crucial to protect the CNS from infections e.g. herpes simplex viruses, varicella zoster virus and JC virus.^26^ However, a dysregulated Tc response can lead to excessive inflammation and neuronal damage.^26^ Tc, especially CD8^+^ Tc, can impair neuronal integrity and excitability causing neuronal degeneration by different mechanisms e.g. granule cytotoxicity mediated by granzyme-B and perforin-, release of cytotoxic cytokines or ligation of death receptors.^12,26^ The pathophysiological relevance of Tc in different diseases is emphasized by their correlation with clinical parameters.^11,22,27^ Apart from the Tc-mediated immune response, antibody-positive ALE shared an expansion of Bc and Pc with RRMS further supporting the relevance of an antigen driven inflammatory response in the immunopathogenesis of antibody-positive ALE.^11^ In turn, antibody-negative ALE patients did not show an excessive Bc and Pc response in the CSF hinting towards a predominantly Tc-mediated immune response similar to TLE-HS and neurodegenerative disorders.^28^ Further, comprehensive immune phenotyping will be needed to enhance the characterization of Bc and Tc populations and interpret their functional relevance in the immunopathogenesis of ALE.

Taking into account clinical manifestation, EEG, MRI and CSF routine parameters, ALE is divided into ‘possible’, ‘probable’ and ‘definite LE’.^1^ However, diagnostic results can sometimes remain inconclusive delaying definitive diagnosis of ALE and treatment initiation. Taking a closer look at the immune cell signature of the ALE cohort, we were able to identify a shared Tc- and/or Bc-driven immune cell profile of ALE irrespective of the ‘likelihood’ of ALE according to the diagnostic consensus criteria^1^ and lateralization on MRI. The shared immune cell profile of definitive and possible/probable ALE indicates that even though the diagnostic consensus criteria offer a reasonable approach to the diagnosis of ALE, they might not be sufficient to allow early diagnosis and to determine the appropriate treatment regime. In the case of antibody-negative ALE, bilateral MRI abnormalities are required to make the diagnosis of definite ALE. However, our results indicate that presence or absence of bilateral MRI abnormalities might not be a suitable parameter for definitive diagnosis of ALE and that diagnosis of ALE should also be considered if no AAB can be detected and MRI abnormalities are unilateral or absent.

Given the shared immune cell profile of ALE, mFC could serve as a complementary diagnostic tool to promote early diagnosis and might affect treatment regimen, thus improving clinical long-term outcomes. With regard to this, our results indicate that novel computational approaches incorporating mFC and CSF routine parameters might be useful to distinguish ALE from clinical differential diagnoses and non-inflammatory controls.

Furthermore, a dysregulated immune response has been reported to negatively impact neuronal integrity and function and to cause neuronal degeneration.^12,26^ Previously described associations between alterations of the immune response (e.g. CD4^+^ and CD8^+^ Tc as well as Pc) and clinical parameters (e.g. cognitive impairment and structural MRI abnormalities) in neurological disorders including ALE emphasize the relevance of immune dysregulation in the pathogenesis of ALE.^11,22^ Accordingly, we found correlations between different innate and adaptive immune cell populations in PB and CSF and cognitive parameters as well as EEG and MRI abnormalities. In CSF, especially higher lymphocyte numbers were associated with poor neuropsychological performance and MRI signal hyperintensities of the amygdalae, which underscores their pathophysiological relevance in ALE. In contrast, PB and CSF NK cells positively correlated with different memory scores supporting their regulatory effects in CNS autoimmune diseases, which is in accordance with previous studies^29-33^ Beyond that, associations between immune cell populations at baseline with clinical parameters at follow-up were noted, which suggests that the immune cell signature at early disease stages might serve as prognostic marker to identify patients benefiting from an early intensified immunomodulatory therapy to improve clinical outcomes.

Moreover, immune cell composition can show dynamic changes over the disease course, which can have clinical and therapeutic implications. However, longitudinal analysis of immune cell profiles of ALE has not been previously performed. Analysing PB and CSF mFC parameters at two different time points we found persisting Tc effector mechanisms at follow-up and a shift from a Bc- and Pc-driven immune response at baseline to a pronounced innate immune response at follow-up. The latter is consistent with the previously described shift from adaptive to innate immunity in multiple sclerosis.^34^ This further emphasizes the crucial role of the Tc immune response during the disease course of ALE and might open up opportunities for novel and individualized treatment regimens depending on the disease stage and underlying immune cell profile. Further longitudinal analyses and an in-depth characterization of different immune cell populations in ALE will be needed to evaluate its pathophysiological and therapeutic relevance.

Our study is limited by the retrospective study design, the imbalanced age and sex ratio between some groups, and the heterogenous follow-up time points. As ALE presents a rare disease entity and patients were recruited over more than 10 years to obtain a sizable patient cohort, no validation of the data was performed in an independent cohort. However, sample collection, processing and mFC analysis were highly standardized and the observations validate previous findings reported in the brain parenchyma of ALE patients.^14,15^ Nevertheless, all findings should be validated in an independent patient cohort in the future to assess the benefit of mFC as complementary tool in the diagnostic workup of ALE. Furthermore, subdivision of the ALE cohort based on the AAB was not performed due to the small sample size in some subcohorts. Thus, future studies should compare ALE patients with different target antigens to assess disease specific changes in the peripheral and intrathecal immune cell profile.

Despite this, one clear strength of our study is the broad characterization of PB and CSF immune cell profiles of a sizable cohort of mainly treatment naive ALE patients—taking into account different clinical parameters and follow-up data—in comparison to various inflammatory and non-inflammatory control groups by combining mFC with novel computational techniques.

## Conclusion

In conclusion, we could identify a pronounced adaptive Bc- and Tc-driven immune response in ALE compared with controls showing similarities in Bc and Tc patterns with classical inflammatory CNS diseases and in Tc patterns with neurodegenerative disorders. Immune cell profiles revealed dynamic changes over time and correlations between immune cell populations and clinical parameters were noted indicating prognostic and therapeutic potential. Beyond that, PB and CSF mFC in combination with CSF routine parameters showed discriminatory power when differentiating ALE from controls and clinical differential diagnosis and thus might present a complementary diagnostic tool to promote early diagnosis of ALE. Further studies are necessary to validate the data in an independent patient cohort to clarify the use of mFC during clinical routine workup.

## Supplementary Material

fcaf156_Supplementary_Data

## Data Availability

Data underlying this study are registered with the ABCD-J data catalogue at https://data.abcd-j.de/dataset/ade1e9cf-f4ad-5f46-9e96-faf9483ae496/1.0. Further information, resources, anonymized clinical and flow cytometry data can be requested via the catalogue item and will be fulfilled by Nico Melzer (Nico.Melzer@med.uni-duesseldorf.de).
