# Supplementary material for: Lymphocyte signatures correspond to clinical phenotypes in autoimmune limbic encephalitis
Source: Brain Commun. 2025 Apr 18;7(2):fcaf156. doi: 10.1093/braincomms/fcaf156 (PMC12038345; doi:10.1093/braincomms/fcaf156)
Supplement: fcaf156_Supplementary_Data [file fcaf156_supplementary_data.pdf]

Supplementary methods

Identification of control cohorts

Patients with relapsing-remitting multiple sclerosis (RRMS; ICD-10 G35.1; n = 148) were diagnosed according to the revised McDonald criteria [1,2]. Extensive diagnostic workup was performed to exclude differential diagnoses as described previously [3]. All patients were treatment-naive at time of sampling. Diagnosis of Alzheimer's disease (AD; ICD-10 G30.9; n = 197) and frontotemporal dementia (FTD; ICD-10 G31.09; n = 67) was made based on the diagnostic criteria by the Institute on Aging and the Alzheimer's Association (NIA-AA) [4] and the diagnostic criteria by the International Behavioral Variant FTD Criteria Consortium (FTDC) [5], respectively. Moreover, patients with temporal lobe epilepsy and hippocampal sclerosis (TLE-HS; ICD-10 G93.81; n = 37), assessed by cranial magnetic resonance imaging (MRI), were included. Patients with somatic symptom disorder (SD; ICD-10 F45; n = 110) were diagnosed according to the ICD-10 diagnostic criteria [6,7] and served as non-inflammatory controls. SD patients had no comorbid neurological conditions and exhibited intrathecal leukocyte counts of <5 cells/µl, intrathecal lactate levels <2 mmol/l, an intact blood-CSF barrier (BCSFB) as indicated by the age-adjusted albumin ratio, no intrathecal immunoglobulin (Ig)-synthesis according to Reiber criteria, and an oligoclonal band pattern type 1, as described previously [8,9].

Patients with chronic infections or comorbid systemic autoimmune disease as well as patients treated with immunosuppressants (except for steroids) prior to sampling were excluded from our study. Parts of the ALE and control cohort were used before [8,9].

The study was performed according to the Declaration of Helsinki and was approved by the local Ethics Committee of the Board of Physicians of the Region Westfalen-Lippe and of the University of Münster, Germany (reference number: 2019-712-f-S). All patients provided written informed consent.

Routine cerebrospinal fluid (CSF) analysis

Lumbar puncture was performed under sterile conditions. Samples were processed immediately (within 1 hour) to ensure optimal sample quality. The number of cells was counted in a Fuchs-Rosenthal chamber. Nephelometry was used to measure total protein and immunoglobulin (Ig) levels (IgG, IgA, and IgM). Oligoclonal bands (ocbs) were detected by isoelectric focusing and silver nitrate staining. Protein and immunoglobulin levels in serum and CSF were analyzed and a Reiber scheme was created to assess the integrity of the blood-CSF barrier (BCSFB) and the quantity of intrathecal Ig synthesis.

Multidimensional flow cytometry

CSF and blood cells (EDTA blood) were analyzed by mFC. Flow cytometry was performed directly on fresh samples. The minimum sample volume was 3 mL. All samples were processed within 1 hour of sampling. The flow cytometer was checked by Flow-Check Fluorospheres (Beckman Coulter) on a daily basis. IMMUNO-TROL cells (Beckman Coulter) were used as reference control to ensure validity of the results. CSF samples were centrifuged for 15 min at 300 g, supernatant was removed, CSF cells were resuspended in parallel to 100µl peripheral blood (PB) in 100 µl VersaLyse (Beckman Coulter) and incubated for 10 min. A washing step was performed and the following fluorochrome-conjugated antibodies, diluted in flow cytometry buffer (FC buffer: PBS/FCS/EDTA) were added: CD3 (UCHT1), CD4 (13B8.2), CD8 (B9.11), CD14 (RM052), CD16 (3G8), CD19 (J3-119), CD45 (J33), CD56 (N901), CD138 (B-A38), and HLA-DR (Immu-357) (Beckman Coulter). Cells were washed, centrifuged, and re-suspended in FC buffer, supplemented with 20 µl flow count fluorospheres (Beckman Coulter). A Navios flow cytometer (Beckman Coulter) was used to acquire data. Data analysis was performed with the software 'Kaluza Flow Cytometry Analysis' version 2.1 (Beckman Coulter). Gates were set by comparing CSF with PB samples ensuring a reliable identification of even small cell populations. The following cell populations were analyzed in

PB and CSF: B cells, monocytes (Mono), classical monocytes (cMono), intermediate monocytes (iMono), non-classical monocytes (ncMono), lymphocytes (Lympho), total T cells, $CD4^+$ T cells ($CD4^+$), $CD4^+CD8^+$ T cells ($CD4^+CD8^+$), $CD4^+HLADR^+$ T cells ($CD4^+HLADR^+$), $CD8^+$ T cells ($CD8^+$), $CD8^+HLADR^+$ T cells ($CD8^+HLADR^+$), plasma cells, $CD4^+/CD8^+$ ratio, granulocytes (Granulo), natural killer cells (NK), natural killer T cells (NKT), $CD56^{dim}$ NK cells (NK dim), and $CD56^{bright}$ NK cells (NK bright). Gating was performed as described previously [8]. First, leukocytes were identified as CD45 positive cells. Next, leukocyte subsets were separated into granulocytes ($SSC^{high}CD14^-$), monocytes ($SSC^{int}CD14^+$), and lymphocytes ($SSC^{low}CD14^-$). Lymphocytes were subdivided into B cells ($CD19^{high}CD138^-$), plasma cells ($CD19^{low}CD138^{high}$), T cells ($CD3^+CD56^-$), which were further classified into $CD4^+$ T cells ($CD4^+CD8^-$), $CD8^+$ T cells ($CD4^-CD8^+$), and $CD4^+CD8^+$ T cells ($CD4^+CD8^+$). In addition, the expression of the activation marker HLA-DR was assessed on $CD4^+$ T cells ($CD4^+HLADR^+$) and $CD8^+$ T cells ($CD8^+HLADR^+$). NK cells were identified as $CD56^+CD3^-$ cells and were divided into $CD56^{bright}$ NK cells ($CD56^{bright}CD16^{dim/-}$) and $CD56^{dim}$ NK cells ($CD56^{dim}CD16^+$). NKT cells were selected as $CD56^+CD3^+$ cells. In addition, monocyte subsets were identified as cMono ($CD14^{high}CD16^-$), iMono ($CD14^+CD16^+$), and ncMono ($CD14^{low}CD16^{high}$) cells. Percentages of PB immune cell populations were compared between groups. Subsequently, the analysis was repeated for CSF immune cell subsets.

Electroencephalography (EEG)

A standard 10-20 system of surface electrode placement was used. To assess medial temporal epileptiform discharges (ED) and slowing, additional anterior temporal electrodes (T1 and T2) were used for short-term EEG recordings and additional basal temporal electrodes FT9/FT10 and TP9/TP10 for long-term EEG recordings. The duration of EEG recordings was between 20 min and 9 days. Standard longitudinal bipolar and common average montages were used for analysis. Presence or absence of interictal ED, slowing, and ictal events according to Graus et.

al. [10] limited to anterior temporal electrodes (F7, F8, T1, T2) were assessed. EEG abnormalities were rated as unilateral if they were confined to the left or right hemisphere. In order to be classified as bilateral (right > left, left > right, left = right), at least one sharp-slow-wave or spike-wave complex had to occur independently to the other hemisphere. Extratemporal ED or slowing were not considered.

Magnetic Resonance Imaging (MRI)

Cranial MRI imaging was performed during clinical routine workup on a 3.0 T- or 1.5 T-MRI scanner at the Department of Radiology of the University Hospital Münster, Germany. T2-FLAIR signal increase of the amygdala and the anterior part of the hippocampus were assessed, indicating medial temporal inflammation. Patients showing diffuse cortico-subcortical T2-FLAIR lesions and gadolinium-enhancing lesions were excluded from the study.

The MRI preprocessing pipeline used to extract Mean T2-weighted fluid-attenuated inversion recovery (FLAIR) Intensities (MFIs) from the FLAIR images is based upon Huppertz et al. [11]: First, the FLAIR image was bias corrected using the "ants.n4_bias_field_correction" method provided by Advanced Normalization Tools in Python (ANTsPy) [12].

Second, an SPM12 [13] pipeline was created containing the following steps:

1. co-registration of the bias corrected FLAIR image to the T1 image
2. spatial normalization of the T1 image
3. spatial normalization of the FLAIR image using normalization parameters of the T1 image

Third, the FLAIR intensity values were normalized using the Fuzzy C-Means (FCM) algorithm [14].

Finally, MFIs were computed using the Automated Anatomical Labeling (AAL) atlas [15] for the amygdala and the atlas provided by Plachti et al. [16] for the anterior hippocampus.

Neuropsychological assessment (NPA)

Visual memory was assessed using the Diagnosticum für Cerebralschädigung II (DCS-II) [17], the Brief Visuospatial Memory Test-Revised (BVMT-R) [18], the CERAD constructional praxis recall [19], and the Rey-Osterrieth Complex Figure Test (RCFT) [20]. Verbal memory was analyzed using the Verbal Learning and Memory Test (VLMT) or the CERAD word list test. For each memory test patient raw scores were transformed into z-scores. A z-score ≤ -1 was considered pathological according to the general convention in neuropsychology [21]. EpiTrack was performed to evaluate attention and executive functions. An EpiTrack score < 29 was considered abnormal. In patients presenting with seizures, NPA was performed after full recovery.

References

1. Polman CH, Reingold SC, Banwell B, Clanet M, Cohen JA, Filippi M, et al. Diagnostic criteria for multiple sclerosis: 2010 revisions to the McDonald criteria. Ann Neurol. 2011 Feb;69(2):292–302.
2. Thompson AJ, Banwell BL, Barkhof F, Carroll WM, Coetzee T, Comi G, et al. Diagnosis of multiple sclerosis: 2017 revisions of the McDonald criteria. Lancet Neurol. 2018 Feb;17(2):162–73.
3. Schafflick D, Xu CA, Hartlehnert M, Cole M, Schulte-Mecklenbeck A, Lautwein T, et al. Integrated single cell analysis of blood and cerebrospinal fluid leukocytes in multiple sclerosis. Nat Commun. 2020 Jan 14;11(1):247.
4. McKhann GM, Knopman DS, Chertkow H, Hyman BT, Jack CR, Kawas CH, et al. The diagnosis of dementia due to Alzheimer’s disease: Recommendations from the National Institute on Aging-Alzheimer’s Association workgroups on diagnostic guidelines for Alzheimer’s disease. Alzheimers Dement. 2011 May;7(3):263–9.

5. Rascovsky K, Hodges JR, Knopman D, Mendez MF, Kramer JH, Neuhaus J, et al. Sensitivity of revised diagnostic criteria for the behavioural variant of frontotemporal dementia. Brain. 2011 Sep;134(Pt 9):2456–77.

6. Buck CJ. 2018 ICD-10-CM Standard Edition - E-Book. Elsevier Health Sciences; 2017.

7. International Statistical Classification of Diseases and Related Health Problems 10th Revision (ICD-10)-WHO Version for;2019-covid-expanded; Chapter VMental and behavioural disorders(F00-F99) [Internet]. [cited 2021 May 4]. Available from: https://icd.who.int/browse10/2019/en#/F40-F48

8. Gross CC, Schulte-Mecklenbeck A, Madireddy L, Pawlitzki M, Strippel C, Räuber S, et al. Classification of neurological diseases using multi-dimensional CSF analysis. Brain. 2021 Oct 22;144(9):2625–34.

9. Lueg G, Gross CC, Lohmann H, Johnen A, Kemmling A, Deppe M, et al. Clinical relevance of specific T-cell activation in the blood and cerebrospinal fluid of patients with mild Alzheimer's disease. Neurobiol Aging. 2015 Jan;36(1):81–9.

10. Graus F, Titulaer MJ, Balu R, Benseler S, Bien CG, Cellucci T, et al. A clinical approach to diagnosis of autoimmune encephalitis. Lancet Neurol. 2016 Apr;15(4):391–404.

11. Huppertz H-J, Wagner J, Weber B, House P, Urbach H. Automated quantitative FLAIR analysis in hippocampal sclerosis. Epilepsy Res. 2011 Nov;97(1–2):146–56.

12. Avants BB, Tustison NJ, Stauffer M, Song G, Wu B, Gee JC. The Insight ToolKit image registration framework. Front Neuroinformatics. 2014 Apr 28;8:44.

13. Friston KJ, Ashburner JT, Kiebel SJ, Nichols TE, Penny WD, editors. Statistical Parametric Mapping: The Analysis of Functional Brain Images. 1st ed. Amsterdam, Boston: Elsevier; 2007.

14. Reinhold JC, Dewey BE, Carass A, Prince JL. Evaluating the impact of intensity normalization on MR image synthesis. Proc SPIE. 2019 Mar;10949.

15. Tzourio-Mazoyer N, Landeau B, Papathanassiou D, Crivello F, Etard O, Delcroix N, et al. Automated Anatomical Labeling (AAL1) atlas. Human Brain Project Neuroinformatics Platform. 2020;
16. Plachti A, Eickhoff SB, Hoffstaedter F, Patil KR, Laird AR, Fox PT, et al. Multimodal parcellations and extensive behavioral profiling tackling the hippocampus gradient. Cereb Cortex. 2019 Dec 17;29(11):4595–612.
17. Weidlich S, Hartje W, Derouiche A, Hillers F. DCS-II: Diagnosticum für Cerebralschädigung - II : ein figuraler visueller Lern- und Gedächtnistest nach F. Hillers. Huber; 2011.
18. Benedict RH. Brief visuospatial memory test--revised. PAR; 1997.
19. Fillenbaum GG, Burchett BM, Unverzagt FW, Rexroth DF, Welsh-Bohmer K. Norms for CERAD constructional praxis recall. Clin Neuropsychol. 2011 Nov;25(8):1345–58.
20. Shin M-S, Park S-Y, Park S-R, Seol S-H, Kwon JS. Clinical and empirical applications of the Rey-Osterrieth Complex Figure Test. Nat Protoc. 2006;1(2):892–9.
21. Iverson GL. Z Scores. In: Kreutzer JS, DeLuca J, Caplan B, editors. Encyclopedia of clinical neuropsychology. New York, NY: Springer New York; 2011. p. 2739–40.

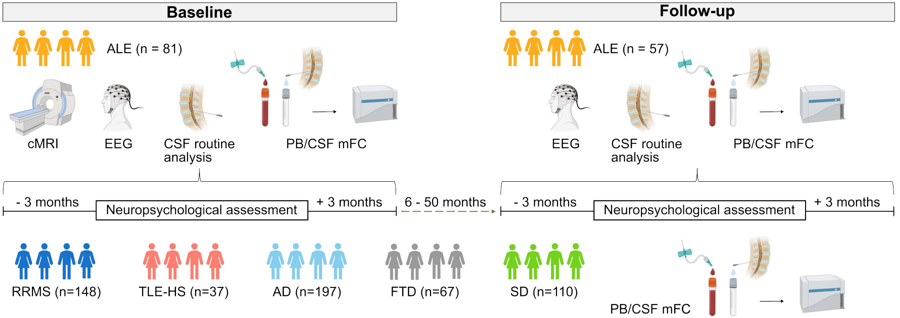

Supplementary figure 1 - Study design

AD:Alzheimer's disease; ALE: autoimmune limbic encephalitis; FTD: frontotemporal dementia; mFC: multidimensional flow cytometry; PB: peripheral blood; RRMS: relapsing-remitting multiple sclerosis; SD: somatic symptom disorder; TLE-HS: temporal lobe epilepsy with hippocampal sclerosis. Created in BioRender. Räuber, S. (2025) https://BioRender.com/g02c503.

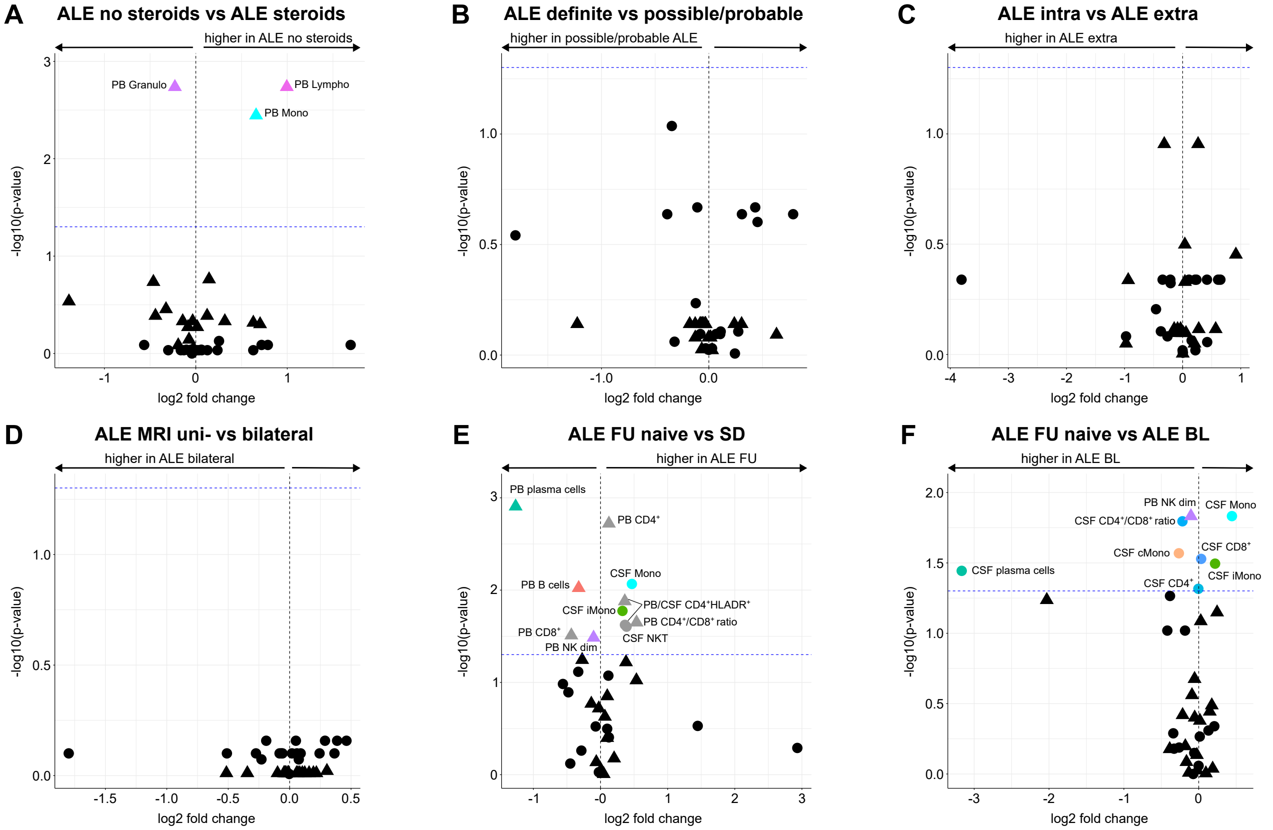

Supplementary figure 2 - Homogenous immune cell profiles amongst ALE subgroups distinct from controls and dynamic changes in immune cell profiles over time

A-F Volcano plots depicting the PB and CSF mFC parameters. The fold change of every single parameter between two groups is plotted against the corresponding p-value calculated by t-test when normality could be assumed based on Shapiro–Wilk test, otherwise Mann–Whitney U test was used. To adjust for multiple comparisons, the Benjamini Hochberg procedure was performed. The following parameters were tested: PB/CSF Mono, PB/CSF cMono, PB/CSF iMono, PB/CSF ncMono, PB/CSF Lympho, PB/CSF B cells, PB/CSF plasma cells, PB/CSF T cells, PB/CSF $CD4^+$, PB/CSF $CD4^+HLADR^+$, PB/CSF $CD8^+$, PB/CSF $CD8^+HLADR^+$, PB/CSF $CD4^+CD8^+$, PB/CSF $CD4^+/CD8^+$ ratio , PB/CSF NK, PB/CSF NK dim, PB/CSF NK bright, PB/CSF NKT, PB/CSF Granulo. PB parameters are shown as triangles and CSF parameters as circles. Only significant parameters are labeled. P-values < 0.0001 are depicted as 0.0001 and p-values > 0.9999 are shown as 1.0.

ALE - autoimmune limbic encephalitis; ALE extra - autoimmune limbic encephalitis with autoantibodies targeting extracellular antigens; ALE intra - autoimmune limbic encephalitis with autoantibodies targeting intracellular antigens; BL - baseline; cMono - classical monocytes; CSF - cerebrospinal fluid; FU - follow-up; Granulo - granulocytes; iMono - intermediate monocytes; Lympho - lymphocytes; mFC - multidimensional flow cytometry; Mono - monocytes; MRI - magnetic resonance imaging; NK - natural killer cells; NKT - Natural killer T cells; PB - peripheral blood; SD - somatic symptom disorder.

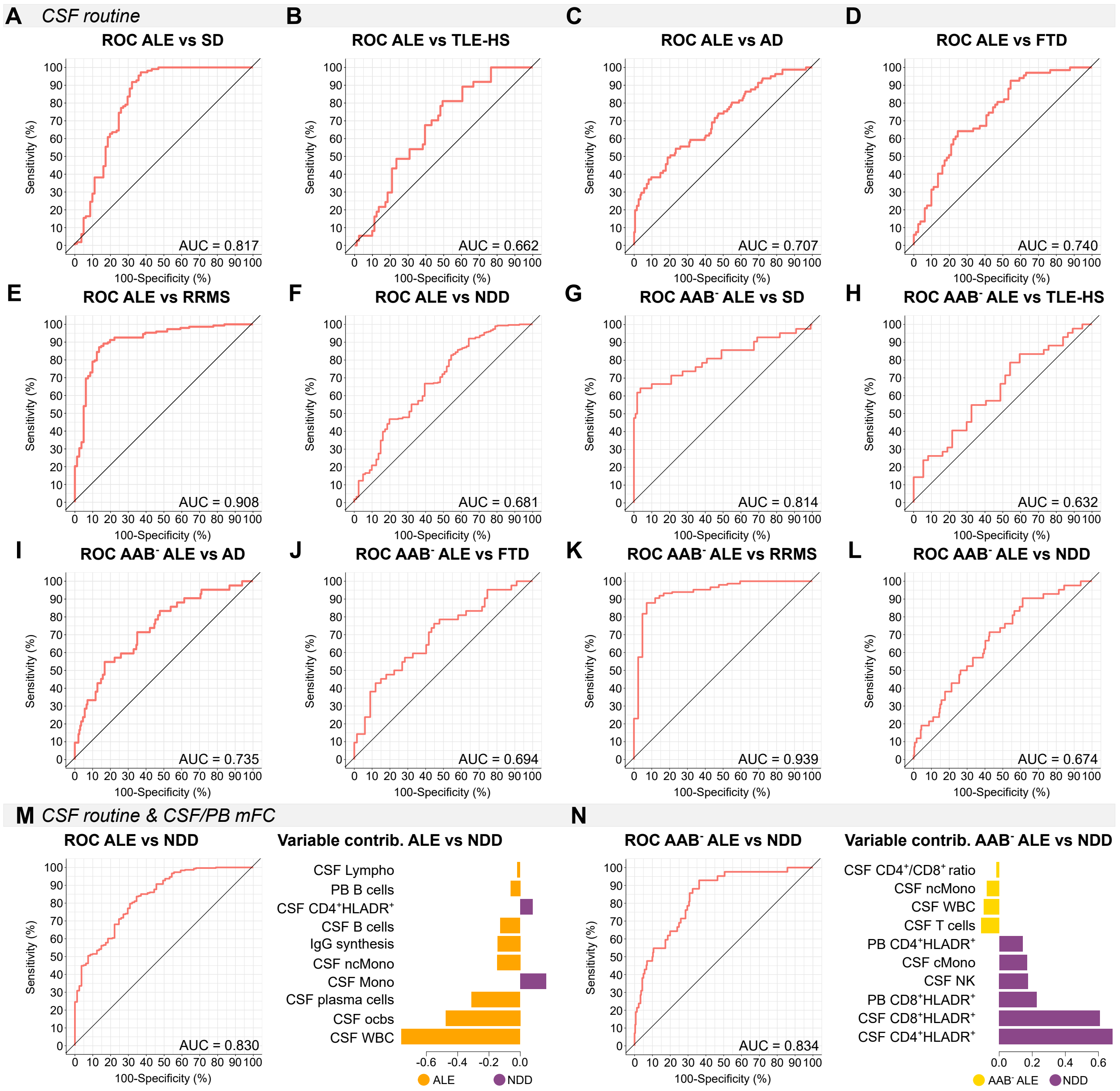

Supplementary figure 3 - CSF routine analysis alone is not suitable to reliably distinguish ALE from clinical differential diagnoses and controls.

A-L ROC analyses of the classification results obtained from sPLS-DA including CSF routine parameters. ALE patients (A-F) or antibody-negative ALE patients (G-L) were compared to one control cohort at a time. **M & N** ROC analyses of the classification results obtained from sPLS-DA. CSF routine as well as PB and CSF mFC parameters were included in the analysis. In addition, the contribution of the top ten variables on latent component 1 was visualized. ALE patients (M) or antibody-negative ALE patients (N) were compared to patients with neurodegenerative disorders (NDD; including AD, FTD, and TLE-HS patients).

AAB$^-$ ALE - autoantibody-negative ALE; AD - Alzheimer's disease; ALE - autoimmune limbic encephalitis; AUC - Area under the curve; cMono - classical monocytes; contrib. - contribution; CSF - cerebrospinal fluid; FTD - frontotemporal dementia; Ig - immunoglobulin; Lympho - lymphocytes; mFC - multidimensional flow cytometry; Mono - monocytes; ncMono - non-classical monocytes; NDD - neurodegenerative disorders; NK - natural killer cells; ocbs - oligoclonal bands; PB - peripheral blood; ROC - receiver operating characteristic; RRMS - relapsing remitting multiple sclerosis; SD - somatic symptom disorder; sPLS-DA - Sparse Partial Least Squares Discriminant Analysis; TLE-HS - temporal lobe epilepsy and hippocampal sclerosis, WBC - white blood cell count.

Supplementary table 1

ID	Sex	Age	Ab	Ab target	Titer CSF	Titer serum	Grauss criteria	Signs and symptoms	Comorbidities	IMD ast	Plex/IA*	Cortisone*	ACD ast	SED ast	Verbal memory (z-score)	Figural memory baseline (z-score)	Epitrack score	EEG abnormal
ALE1	f	27	-	-	-	-	definite	memory and attention deficits, seizures, emotional instability, depression	no	no	no	no	yes	no	2.39	-0.15	33	yes
ALE2	f	64	DPPX	extra	1:1	1:1000	definite	memory deficits, autonomic dysfunction, diarrhea, weight loss, nystagmus, gait disturbance, vertigo, diplopia, depression	diabetes, hypothyroidism	no	no	no	no	no	1.14	na	na	no
ALE3	f	23	-	-	-	-	definite	memory deficits, seizures	s/p ALL	no	no	yes	yes	no	na	-1.25	36	yes
ALE4	m	80	Hu	intra	1:100	1:1000	possible	memory deficits, disorientation, depression, speech disturbances, seizures	SCLC, afib, hypertension, PAD	no	no	no	no	no	-3.09	na	13	yes
ALE5	m	47	GAD65	intra	1:100	1:1000	possible	memory deficits, seizures, emotional instability, anxiety	adrenal adenoma, hypertension, obesity, OSAS	no	no	no	yes	no	-0.30	0.22	35	no
ALE6	m	65	CASPR2	extra	1:320	1:3200	possible	memory deficits, disorientation, seizures, formal thought disorder, infringing personal space	CTS, s/p stroke, diabetes, hypertension, COPD	no	no	no	yes	no	-2.49	-0.85	19	yes
ALE7	m	67	-	-	-	-	definite	memory deficits, seizures, depression	PNP, hypertension	no	no	no	no	no	-1.41	na	na	yes
ALE8	f	17	-	-	-	-	possible	seizures, depression	hypothyroidism	no	no	no	yes	no	0.84	na	41	yes
ALE9	f	48	-	-	-	-	definite	memory deficits, disorientation, psychomotor retardation, seizures, psychosis, autonomic dysfunction	hypothyroidism, hypertension, bipolar disorder, obesity	no	no	yes	yes	no	-3.59	na	16	yes
ALE10	m	66	-	-	-	-	probable	memory deficit, psychomotor retardation, depression	bipolar disorder, diabetes, hypertension, PNP	no	no	no	no	no	na	na	34	no
ALE11	f	53	GAD65	intra	1:10	1:320	definite	memory deficits, seizures	diabetes, rosacea	no	no	no	yes	no	-0.17	na	40	no
ALE12	m	53	-	-	-	-	probable	memory deficits, depression	hypothyroidism	no	no	no	no	yes	-1.75	na	34	yes
ALE13	m	50	LGI1	extra	0	1:32	possible	memory deficits, disorientation, seizures, anxiety, infringing personal space	asthma	no	no	no	yes	no	-1.88	-2.23	26	yes
ALE14	m	41	GAD65	intra	1:3.2	1:1000	possible	memory deficits, disorientation, seizures	no	no	no	no	yes	no	0.71	-0.45	34	yes
ALE15	m	44	-	-	-	-	definite	memory deficits, seizures	no	no	no	no	yes	no	0.20	0.22	40	yes
ALE16	f	49	unknown	unknown	1:320	0	possible	memory deficits, seizures, depression	PNP, hashimoto thyroiditis, migraine, hypertension	no	no	no	yes	no	0.84	-2.26	35	yes

ID	Sex	Age	Ab	Ab target	Titer CSF	Titer serum	Grauss criteria	Signs and symptoms	Comorbidities	IMD ast	Plex/IA*	Cortisone*	ACD ast	SED ast	Verbal memory (z-score)	Figural memory baseline (z-score)	Epitrack score	EEG abnormal
ALE17	m	64	-	-	-	-	definite	memory deficits, disorientation, seizures	s/p PE	no	no	yes	yes	no	-1.08	na	37	no
ALE18	f	59	Hu	intra	1:320	1:10000	definite	depression, anxiety, hallucinations	COPD, hypertension	no	no	no	no	yes	-0.77	-2.33	28	no
ALE19	m	52	-	-	-	-	definite	seizures, depression	asthma	no	no	no	yes	no	1.74	-0.93	38	no
ALE20	m	67	-	-	-	-	definite	memory deficit, seizures, depression	s/p lymphoma, s/p testicular cancer	no	no	no	yes	no	0.84	-0.50	36	yes
ALE21	m	70	-	-	-	-	possible	memory deficits, seizures, impulsive behavior, depression	s/p stroke, hypertension	no	no	no	no	no	-0.47	-0.25	25	yes
ALE22	m	53	-	-	-	-	definite	memory deficits, seizures, autonomic dysfunction	no	no	no	no	no	no	-0.88	-0.64	31	no
ALE23	f	43	GAD65	intra	1:10	1:100	definite	memory deficits, seizures, depression	no	no	no	no	no	no	1.73	-1.23	33	yes
ALE24	m	51	GABA-A-R	intra	0	1:10	definite	memory deficits, seizures, psychosis, psychomotor retardation	s/p stroke, hypertension, adrenal adenoma, OSAS, asthma	no	yes	no	yes	no	-1.75	-1.07	na	yes
ALE25	f	61	-	-	-	-	possible	memory deficits, seizures	hypertension, hypothyroidism, afib, OSAS, buccal mucosa cancer	no	no	no	no	no	-0.47	-1.26	30	yes
ALE26	m	74	LGI1	extra	0	1:1000	definite	memory deficits, disorientation, seizures, speech disturbances, autonomic dysfunction, apraxia	CAD, s/p TIA, VaD, diabetes, hypertension	no	no	no	no	no	-4.22	-4.25	na	yes
ALE27	m	58	-	-	-	-	possible	seizures	depression, PNP	no	no	no	yes	no	-0.57	-1.10	35	yes
ALE28	f	60	GAD65	intra	1:100	1:100	possible	memory deficits, seizures, depression	s/p thymoma, hypertension, meningioma	no	no	no	yes	no	-0.07	-1.33	36	yes
ALE29	m	45	-	-	-	-	possible	memory deficits, seizures	hypertension	no	no	no	no	no	0.71	-0.29	36	yes
ALE30	f	67	GAD65	intra	1:1	1:1000	definite	memory deficits, psychomotor retardation, emotional instability, seizures	s/p hashimoto thyroiditis, vitiligo	no	no	no	na	na	-0.17	na	37	yes
ALE31	f	56	GABA-B-R	extra	0	1:10	definite	seizures	ITP	no	no	no	yes	no	-0.37	0.18	37	yes
ALE32	f	22	-	-	-	-	definite	seizures	depression	no	no	no	yes	no	2.12	1.48	33	yes
ALE33	m	59	-	-	-	-	possible	memory deficits, seizures, psychosis, impulsive behavior, speech disturbances	OSAS, depression	no	no	no	yes	no	0.64	na	35	yes
ALE34	m	33	GAD65	intra	1:3.2	1:100	possible	psychomotor retardation, seizures, emotional instability, depression	chronic sinusitis	no	no	yes	yes	no	-1.83	-0.98	22	no
ALE35	m	66	CASPR2	extra	1:320	1:3200	possible	memory deficits, seizures	PAD, hypothyroidism	no	no	no	yes	no	-0.27	-2.12	32	yes

ID	Sex	Age	Ab	Ab target	Titer CSF	Titer serum	Grauss criteria	Signs and symptoms	Comorbidities	IMD ast	Plex/IA*	Cortisone*	ACD ast	SED ast	Verbal memory (z-score)	Figural memory baseline (z-score)	Epitrack score	EEG abnormal
ALE36	m	53	-	-	-	-	definite	memory deficits, psychomotor retardation, depression, personality changes	hypertension, diabetes, obesity, COPD, OSAS, cardiomyopathy, hypercholesterolemia	no	no	no	no	no	0.64	-0.99	31	no
ALE37	m	53	-	-	-	-	definite	memory deficits, seizures	no	no	no	no	na	na	-2.28	-2.23	26	yes
ALE38	f	49	-	-	-	-	definite	memory deficits	hypertension	no	no	no	yes	no	2.49	-0.78	40	yes
ALE39	m	54	CASPR2	extra	1:320	1:3200	definite	memory deficits, seizures	no	no	no	no	no	no	-0.57	na	32	yes
ALE40	f	55	-	-	-	-	possible	memory deficits, seizures, depression	PLMS	no	no	no	yes	no	0.53	-1.40	35	yes
ALE41	m	44	-	-	-	-	definite	seizures, impulsive behavior, depression	thrombophilia, s/p PME and DVT, s/p testicular cancer, aortic stenosis	no	no	no	yes	no	0.08	-0.24	36	yes
ALE42	f	59	GAD65	intra	0	1:100	definite	memory deficits, seizures, infringing personal space, depression	chronic headache, s/p PME and DVT, cholecystolithiasis, urolithiasis	no	no	no	yes	no	0.23	na	na	yes
ALE43	f	61	-	-	-	-	possible	memory deficits, emotional instability, depression	chronic tension-type headache	no	no	no	no	no	0.53	-1.44	na	yes
ALE44	m	64	unknown	unknown	0	1:1000	definite	memory deficits, psychomotor retardation, seizures, emotional instability, depression, apraxia	CAD, emphysema, s/p duodenal ulcers	no	no	no	no	no	-0.57	0.08	31	no
ALE45	f	71	LGI1	extra	0	1:100	definite	memory deficits, seizures, autonomic dysfunction	CAD, diabetes, hypertension, hypothyroidism	no	no	no	yes	no	-1.88	na	na	yes
ALE46	m	49	LGI1	extra	1:1	1:1000	definite	memory deficits, seizures	no	no	no	yes	no	no	0.00	0.22	40	yes
ALE47	m	75	-	-	-	-	definite	memory deficits, psychomotor retardation, seizures, personality changes	PNP	no	no	no	no	no	0.33	-0.81	35	yes
ALE48	f	76	LGI1	extra	1:1	1:1000	possible	memory deficits, seizures, depression, personality changes	hypertension, goiter	no	no	no	yes	no	-0.37	na	25	yes
ALE49	m	56	GABA-B-R	extra	0	1:10	definite	memory deficits, psychomotor retardation, seizures, infringing personal space	no	no	no	no	yes	no	-0.98	-0.04	31	no
ALE50	m	58	-	-	-	-	definite	memory deficits, psychomotor retardation, seizures, agitation, anxiety, depression	PNP, CAD, PAD	no	no	no	yes	no	0.03	-1.76	34	no
ALE51	m	22	-	-	-	-	definite	seizures	atopic dermatitis	no	no	no	no	no	-0.26	-2.75	32	yes

ID	Sex	Age	Ab	Ab target	Titer CSF	Titer serum	Grauss criteria	Signs and symptoms	Comorbidities	IMD ast	Plex/IA*	Cortisone*	ACD ast	SED ast	Verbal memory (z-score)	Figural memory baseline (z-score)	Epitrack score	EEG abnormal
ALE52	m	66	LGI1	extra	1:1	1:100	possible	memory deficits, disorientation, impulsive behavior, psychosis, apraxia	Parkinson disease, Graves disease	no	no	yes	no	yes	-3.29	-4.25	20	yes
ALE53	f	71	-	-	-	-	possible	memory deficits, seizures, impulsive behavior, anxiety, autonomic dysfunction	hypertension, afib, goiter, renal angiomyolipoma	no	no	no	yes	no	-1.28	-2.33	24	yes
ALE54	m	78	LGI1	extra	1:1	1:100	definite	memory deficits, disorientation, psychomotor retardation, emotional instability, seizures, speech disturbances	hypertension, OSAS, heart failure	no	no	yes	yes	yes	-2.28	na	24	no
ALE55	f	68	-	-	-	-	possible	seizures, impulsive behavior, anxiety	s/p DVT	no	no	yes	yes	no	-1.18	na	26	yes
ALE56	f	61	-	-	-	-	possible	seizures, emotional instability, depression, speech disturbances	hypothyroidism	no	no	no	yes	yes	-1.68	-1.94	22	yes
ALE57	f	35	GAD65	intra	na	na	definite	memory deficits, seizures, depression, personality changes, speech disturbances	hypothyroidism, diabetes	no	no	no	yes	no	0.20	0.71	38	yes
ALE58	m	63	MaTa	intra	1:10	1:10	definite	memory deficits, disorientation, personality changes, depression, apraxia	heart failure, s/p PME and DVT, COPD, diabetes, hypertension, fatty liver	no	no	yes	yes	yes	-3.28	-2.20	na	yes
ALE59	m	41	VGKC	extra	0	1:64	definite	memory deficits, seizures, infringing personal space	amblyopia, strabismus, cataract	no	no	no	yes	no	-1.83	0.81	37	yes
ALE60	f	57	GAD65	intra	1:10	1:1000	definite	memory deficits, seizures	PNP, hypertension	no	no	no	yes	no	-1.75	-1.63	21	yes
ALE61	m	53	Neurexin-3α	extra	1:1	1:100	definite	memory deficits, disorientation, seizures, depression, psychosis, parkinsonism	s/p DVT, hypertension	no	no	no	no	yes	-1.88	-1.73	31	no
ALE62	m	61	-	-	-	-	definite	memory deficits, seizures, depression	no	no	no	no	no	no	0.84	-0.38	na	yes
ALE63	m	71	Hu, Sox Zic4, Yo	intra	na	na	possible	memory deficits, disorientation, agitation, seizures, ataxia, hypoesthesia, dysesthesia	SCLC, COPD, urothelial carcinoma	no	no	yes	yes	no	-1.38	na	na	yes
ALE64	f	74	Ma2	intra	na	na	possible	memory deficits, impulsive behavior, psychomotor retardation, speech disturbances	s/p breast cancer, goiter, osteoporosis, pituitary adenoma	no	no	no	yes	no	-2.97	-2.25	26	yes
ALE65	f	64	-	-	-	-	possible	memory deficits, disorientation, seizures, depression, speech disturbances	s/p stroke, PNP, CAD, hypertension	no	no	no	yes	no	-0.17	na	32	yes

ID	Sex	Age	Ab	Ab target	Titer CSF	Titer serum	Grauss criteria	Signs and symptoms	Comorbidities	IMD ast	Plex/IA*	Cortisone*	ACD ast	SED ast	Verbal memory (z-score)	Figural memory baseline (z-score)	Epitrack score	EEG abnormal
ALE66	f	63	LGI1	extra	na	1:100	definite	memory deficits, disorientation, seizures, infringing personal space, formal thought disorder	no	no	no	yes	yes	yes	-1.18	-1.84	13	no
ALE67	f	54	LGI1	extra	0	1:10	definite	memory deficits, seizures, depression, personality changes	ovarian cancer, hypertension	no	no	yes	no	no	1.14	-0.24	38	no
ALE68	m	74	-	-	-	-	definite	memory deficits, depression, hypoesthesia/paranesthesia	s/p stroke, s/p herpes zoster, hypertension, s/p MI, OSAS, MUGS	no	no	no	no	no	-0.07	-0.60	35	no
ALE69	f	68	-	-	-	-	definite	memory deficits, psychomotor retardation, seizures	s/p encephalitis of unknown origin, depressive episode, PNP, CTS, hypertension, goiter, s/p herpes zoster	no	no	no	yes	no	-0.98	na	24	yes
ALE70	m	69	LGI1	extra	1:1	1:320	definite	memory deficits, disorientation, seizures, depression, speech disturbances, autonomic dysfunction	CAD, hypertension	no	no	no	yes	no	-0.17	-1.40	38	no
ALE71	m	53	-	-	-	-	probable	psychomotor retardation, speech disturbances	hypertension, s/p acute hepatitis B, s/p testicular cancer, CKid	no	no	no	no	no	-0.67	-2.33	34	yes
ALE72	f	48	-	-	-	-	possible	memory deficits, psychomotor retardation, seizures, depression, impulsive behavior, skew deviation	s/p stroke	no	no	no	yes	no	-1.96	-2.33	22	yes
ALE73	f	50	-	-	-	-	definite	memory deficits, seizures, infringing personal space, formal thought disorder, speech disturbances	PNP, s/p DVT, asthma, migraine, chronic gastritis, colorectal adenomas	no	no	no	yes	no	-0.47	-2.33	34	yes
ALE74	f	56	LGI1	extra	1:100	1:320	possible	memory deficits, disorientation, seizures, impulsive behavior, confabulations	s/p DVT	no	no	no	yes	no	-5.47	-2.75	na	yes
ALE75	m	76	DPPX	extra	1:32	1:10000	definite	memory deficits, disorientation, infringing personal space, depression, parkinsonism, gait disturbance	hypertension, s/p RCC	no	no	no	no	yes	-2.49	na	20	no
ALE76	f	21	Yo, Ma2/Ta	intra	na	na	definite	memory deficits, psychomotor retardation, seizures, infringing personal space	no	no	no	no	yes	no	2.12	0.22	40	yes

ID	Sex	Age	Ab	Ab target	Titer CSF	Titer serum	Grauss criteria	Signs and symptoms	Comorbidities	IMD ast	Plex/IA*	Cortisone*	ACD ast	SED ast	Verbal memory (z-score)	Figural memory baseline (z-score)	Epitrack score	EEG abnormal
ALE77	m	57	-	-	-	-	definite	memory deficits, seizures, impulsive behavior, emotional instability	no	no	no	no	yes	no	-0.47	-0.45	35	yes
ALE78	m	61	-	-	-	-	definite	seizures, speech disturbances	AVNRT, s/p TIA, afib, hypertension, hypothyroidism	no	no	no	no	no	0.03	2.25	36	yes
ALE79	f	29	GFAP	intra	1:32	1:100	possible	memory deficits, formal thought disorder	fibromyalgia	no	no	yes	yes	no	0.00	-1.25	25	yes
ALE80	m	43	GAD65	intra	1:10	1:1000	definite	memory deficits, seizures, autonomic dysfunction	asthma, s/p Graves disease	no	no	yes	yes	no	0.20	2.07	na	no
ALE81	m	27	-	-	-	-	definite	memory deficits, seizures, depression	s/p thyroid cancer, s/p depressive episode, obesity	no	no	no	yes	no	1.20	0.71	38	yes

Baseline disease characteristics of ALE patients

Ab - antibody; ACD - anticonvulsive drugs; afib - atrial fibrillation; ALE - autoimmune limbic encephalitis; AVNRT - AV nodal reentrant tachycardia; CAD - coronary artery disease; CKid - chronic kidney disease; cMRI - cranial magnetic resonance imaging; COPD - Chronic obstructive pulmonary disease; CTS - carpal tunnel syndrome; DVT - deep vein thrombosis; EEG - electroencephalography; extra - extracellular; IA - immunoadsorption; intra - Intracellular; IMD - immunomodulatory drug; ITP - isolated thrombocytopenia; MI - myocardial infarction; MUGS - monoclonal gammopathy of undetermined significance; OSAS - Obstructive sleep apnea syndrome; PAD - peripheral artery disease; PLEX - plasmapheresis; PLMS - Periodic limb movements of sleep; PME - pulmonary embolism; PNP - polyneuropathy; RCC - renal cell carcinoma; SCLC - small cell lung cancer; SED - sedative drugs; s/p - status post; TIA - Transient ischemic attack; VaD - vascular dementia

** within 4 weeks before sample taking*

Supplementary table 2

mFC & clinical data baseline

A

	PB mFC absolute							
	iMono	Lympho	Tc	$CD8^+$	$CD8^+HLADR^+$	NK	NK dim	NK bright
NPA figural memory (z-score)	ns	ns	ns	ns	ns	0.311	0.315	0.260
NPA verbal memory (z-score)	-0.242	ns	ns	ns	ns	0.243	0.268	ns
NPA EpiTrack score	ns	0.286	0.290	0.392	0.247	0.358	0.351	0.340
EEG interictal EDs/slowing	ns	ns	ns	ns	ns	ns	ns	ns
MRI MFI amygdalae (mean)	ns	ns	ns	ns	ns	ns	ns	ns
MRI MFI hippocampi (mean)	ns	ns	ns	ns	ns	ns	ns	ns

	PB mFC relative									
	cMono	iMono	ncMono	Granulo	NK	Lympho	$CD4^+$	$CD8^+$	$CD4^+/CD8^+$	Pc
NPA figural memory (z-score)	ns	ns	ns	ns	ns	ns	ns	ns	ns	-0.281
NPA verbal memory (z-score)	0.228	-0.267	ns	ns	0.250	0.241	-0.243	ns	ns	-0.229
NPA EpiTrack score	0.319	-0.333	-0.263	-0.341	ns	0.360	-0.352	0.354	-0.371	ns
EEG interictal EDs/slowing	ns	ns	ns	ns	ns	ns	ns	ns	ns	ns
MRI MFI amygdalae (mean)	ns	ns	ns	ns	ns	ns	ns	ns	ns	ns
MRI MFI hippocampi (mean)	ns	ns	ns	ns	ns	ns	ns	ns	ns	ns

B

	CSF routine		CSF mFC absolute									
	Protein	WBC	Lympho	Tc	$CD4^+$	$CD4^+HLADR^+$	$CD8^+$	$CD8^+HLADR^+$	$CD4^+CD8^+$	Bc	Pc	Granulo
NPA figural memory (z-score)	ns	-0.328	-0.315	-0.314	-0.328	-0.276	-0.283	ns	ns	ns	-0.308	0.278
NPA verbal memory (z-score)	ns	-0.305	ns	ns	ns	ns	-0.223	-0.231	-0.235	-0.230	-0.332	ns
NPA EpiTrack score	ns	-0.377	ns	ns	ns	ns	ns	ns	ns	ns	-0.292	ns
EEG interictal EDs/slowing	ns	ns	ns	ns	ns	ns	ns	ns	ns	ns	ns	ns
MRI MFI amygdalae (mean)	0.300	ns	ns	ns	ns	ns	ns	ns	ns	ns	0.380	ns
MRI MFI hippocampi (mean)	ns	ns	ns	ns	ns	ns	ns	ns	ns	ns	ns	ns

	CSF mFC relative					
	ncMono	NK	Lympho	$CD4^+CD8^+$	Bc	Pc
NPA figural memory (z-score)	ns	ns	-0.371	ns	ns	-0.316
NPA verbal memory (z-score)	-0.228	ns	ns	ns	ns	-0.329
NPA EpiTrack score	ns	ns	ns	ns	ns	-0.269
EEG interictal EDs/slowing	ns	ns	ns	-0.240	ns	ns
MRI MFI amygdalae (mean)	ns	ns	ns	ns	0.276	0.330
MRI MFI hippocampi (mean)	ns	-0.268	ns	ns	ns	ns

C

	CSF routine	CSF mFC absolute								
	WBC	Lympho	Tc	$CD4^+$	$CD4^+HLADR^+$	$CD8^+$	$CD8^+HLADR^+$	$CD4^+CD8^+$	Bc	Pc
AAB titre serum	ns	ns	ns	ns	ns	ns	ns	ns	ns	ns
AAB titre CSF	0.467	0.411	0.397	0.418	0.457	0.366	0.358	0.345	0.513	0.547

	CSF mFC relative						MRI
	Mono	Granulo	NKT	Lympho	Bc	Pc	MRI MFI amygdalae (mean)
AAB titre serum	ns	ns	-0.478	ns	ns	ns	ns
AAB titre CSF	-0.437	-0.483	-0.574	0.569	0.414	0.478	0.500

mFC data baseline & clinical data follow-up

D	PB mFC absolute					PB mFC relative			CSF routine	CSF mFC relative		
	**NK**	**NK dim**	**NK bright**	**Lympho**	**$CD8^+$**	**NK**	**$CD8^+HLADR^+$**	**Bc**	**Protein**	**NK dim**	**NK bright**	**$CD4^+HLADR^+$**
NPA figural memory (z-score)	ns	ns	ns	ns	ns	0.405	ns	0.370	ns	ns	ns	ns
NPA verbal memory (z-score)	0.437	0.393	0.480	0.569	0.385	ns	-0.403	ns	ns	0.471	-0.350	-0.390
NPA EpiTrack score	ns	ns	ns	ns	ns	ns	ns	ns	ns	ns	ns	ns
EEG interictal EDs/slowing	ns	ns	ns	ns	ns	ns	ns	ns	0.448	ns	ns	ns

Immune cell signature of ALE correlate with clinical parameters

Correlation analysis was performed with Pearson correlation coefficient (if normality could be assumed based on D'Agostino & Pearson test) or Spearman correlation (if no normal distribution could be assumed): A Correlation between PB mFC parameters (absolute and relative numbers) at baseline and NPA performance, EEG abnormalities, and MRI MFI values at baseline; B Correlation between CSF routine and CSF mFC parameters (absolute and relative numbers) at baseline and NPA performance, EEG abnormalities, and MRI MFI values at baseline; C Correlation between AAB titres in serum and CSF routine and PB/CSF mFC parameters (absolute and relative numbers) as well as MRI MFI values; D Correlation between CSF routine, PB/CSF mFC parameters (absolute and relative numbers) at baseline and NPA performance and EEG abnormalities at follow-up.

AAB - autoantibody; Bc - B cells; cMono - classical monocytes; CSF - cerebrospinal fluid; EDs - epileptiform discharges; EEG - electroencephalography; Granulo - granulocytes; iMono - intermediate monocytes; Lympho - lymphocytes; mFC - multidimensional flow cytometry; MFI - Mean FLAIR Intensity; Mono - monocytes; MRI - magnetic resonance imaging; ncMono; non-classical monocytes; NK - natural killer cells; NKT - Natural killer T cells; NPA - neuropsychological assessment; ns - not significant; PB - peripheral blood; Pc - plasma cells; Tc - T cells; WBC - white blood cell count.
